# Supplementary material for: Improving fragment-based ab initio protein structure assembly using low-accuracy contact-map predictions
Source: Nat Commun. 2021 Aug 18;12:5011. doi: 10.1038/s41467-021-25316-w (PMC8373938; doi:10.1038/s41467-021-25316-w)
Supplement: Supplementary file 1 — Supplementary Information [file 41467_2021_25316_MOESM1_ESM.pdf]

SUPPLEMENTARY INFORMATION

Table of Content

SUPPLMENTARY TEXTS

Text S1. Contact selection based on effective number of homologous sequences and protein lengths
Text S2. Confidence score cut-offs for each contact predictor when selecting contacts
Text S3. C-QUARK force field used to guide the REMC simulations
Text S4. Well depth in the contact potential

SUPPLEMENTARY TABLES

Table S1: Average accuracies of all-range and long-range predicted contacts used in C-QUARK
Table S2: Average accuracies of the different ranges of predicted contacts used in C-QUARK
Table S3: Average satisfaction rates of different ranges of contacts in the first predicted models
Table S4: Average TM-scores and GDT_TS scores for the first models generated by C-QUARK and the control versions of the C-QUARK pipeline.
Table S5: Average TM-scores, GDT_TS scores, and RMSDs for the first models generated by C-QUARK and the control methods on the 247 test proteins.
Table S6: Average TM-scores, GDT_TS scores, and RMSDs for the first models generated by C-QUARK and the control methods on the 59 test proteins with low contact-map prediction accuracy.
Table S7: Average TM-scores, GDT_TS scores, and RMSDs for the first models generated by C-QUARK and control methods on the 57 non-redundant test proteins.
Table S8: Summary of C-QUARK on the 64 targets compared with the top five servers in CASP13
Table S9: Summary of C-QUARK on the 64 CASP13 targets compared with distance-based folding methods.
Table S10: Performance of C-QUARK on 21 CASP13 multi-domain proteins.
Table S11: Top *L* contact prediction accuracy for different predictors and the consensus
Table S12: Top *L*/2 contact prediction accuracy for different predictors and the consensus
Table S13: Top *L* contact prediction accuracy for different predictors on the training proteins
Table S14: Top *L*/2 contact prediction accuracy for different predictors on the training proteins
Table S15: Width of the first well (d_b) in the contact potential at various protein lengths (L)
Table S16: Minimum number of contacts at different protein lengths (L) and N_f of MSA
Table S17: Confidence score cut-offs of different contact predictors

SUPPLEMENTARY FIGURES

Figure S1: RMSD comparison between C-QUARK and QUARK.
Figure S2: TM-scores of C-QUARK and QUARK at different protein lengths.
Figure S3: Accuracies of predicted contacts versus those derived from the final models.
Figure S4: Confidence scores versus long-range accuracies by different programs
Figure S5: Contact satisfaction rate versus contact prediction accuracy.
Figure S6: Effect of contact satisfaction rate on the TM-scores of the first C-QUARK models.
Figure S7: A representative case showing contact satisfaction rate during the simulation trajectories.
Figure S8: An example illustrating how contact restraints help C-QUARK modeling.
Figure S9: TM-score comparison between C-QUARK and the control methods for 247 test proteins and its subsets.
Figure S10: Contact accuracy versus N_f for the cases that are not foldable by C-QUARK.
Figure S11: Case studies on proteins not folded by C-QUARK although they had reasonable contact-maps.
Figure S12: Case study on CASP13 FM target, T0980s1-D1.
Figure S13: Dependence of C-QUARK run time on protein length.

Figure S14: Boxplot and distribution for TM-scores of the first models produced by C-QUARK on 21 CASP13 multi-domain targets and the corresponding 62 individual domains.
Figure S15: TM-score comparison of C-QUARK using all ten contact predictors vs using ResPRE alone.
Figure S16: Eleven local movements in the C-QUARK REMC folding simulations.
Figure S17: A schematic of the contact potential used in C-QUARK.
Figure S18: Correlation of contacts with N_f.
Figure S19: Correlation of TM-score with the contact accuracy derived from the final models.

SUPPLEMENTARY REFERENCES

SUPPLEMENTARY TEXTS

Text S1. Contact selection based on effective number of homologous sequences (N_f) and protein length

According to the performance shown in Table S13 and S14, based on the training proteins (Dataset S1 in SI), the contact predictors are classified into four categories: *i*) NeBcon [1], ResPRE [2] and DeepPLM as "very high", *ii*) DeepCov [3], Deepcontact [4] and DNCON2 [5] as "high", *iii*) MetaPSICOV2 [6] as "medium, and *iv*) GREMLIN [7], CCMpred [8] and FreeContact [9] as "low". We consider the effective number of sequences (N_f) in the corresponding multiple sequence alignment (MSA) and length of the target as criteria when selecting the number of contacts from each of these categories. Here, N_f is defined by

$$N_f = \frac{1}{\sqrt{L}} \sum_{i=1}^{nseq} \frac{1}{1 + \sum_{j=1, j\neq i}^{nseq} I\left[S_{ij} \geq s\right]} \tag{S1}$$

where L is the length of the protein and *nseq* is the total number of sequences in an MSA. S_{ij} is the sequence identity between sequence *i* and sequence *j* in an MSA, and *s*=0.80 is the sequence identity cut-off . *I*[ ] is the Iverson bracket, i.e $I\left[S_{ij} \geq s\right]$=1 if $S_{ij} \geq s$, and 0 otherwise.

For instance, at least the top *L, L/2, L/4.5* and *L/7.5* contacts are selected from the different confidence score categories, regardless of the length of the target, when N_f <50. On the other hand, if N_f is ≥50 and the length of the target is <120, at least the top *L/2, L/3, L/5.5* and *L/8.5* contacts are selected from these categories. The rational for selecting fewer contacts for the latter condition is twofold: *i*) small-sized proteins usually have less contacts, and *ii*) the contact prediction accuracy increases with an increase in the N_f value, as shown in Figs S18A and S18B, and hence fewer high-resolution contacts are necessary as restraints to produce successfully folded models. On the other hand, although the contact prediction accuracy is usually low at relatively lower N_f values, often the predicted contacts are very near to the true contacts. As a result, selecting more contacts in the former condition may capture the overall 3D contact network and possibly be helpful in modeling, since other energy potential terms help to eliminate the wrong contacts anyway as demonstrated in Fig. 4. Finally, when the length of the target is >=120 with N_f >50, more contacts are needed so that the restraints from the contacts can be imposed throughout the sequence. Therefore, at least the top *L/1.25, L/2.25, L/4.75* and *L/7.75* contacts are selected from the different categories. Table S16 summarizes the number of contacts selected from the different categories that were obtained based on several trials of training using the training proteins.

Text S2. Confidence score cut-offs for each contact predictor when selecting contacts

In addition to the above mentioned criteria, we also consider an accuracy threshold for each predictor, where contacts with accuracies greater than the threshold are selected from each of the predictors. However, it is not possible to know the accuracy of predicted contacts without prior knowledge of the corresponding native structure. One solution to estimate the accuracy of predicted contacts is based on the confidence score of contacts between residue pairs, since the confidence score has a strong correlation with the accuracy of the contacts, as shown in Fig. S4. However, due to the variation of scoring schemes in different contact predictors, the correlation is often not linear. Therefore, we choose different confidence score cut-offs for different predictors that correspond to at least a contact accuracy of 0.5, as shown with a dashed line in Fig. S4 for long-range contacts. The confidence cut-offs corresponding to an accuracy of 0.5 are summarized in Table S1 for different range contacts. The consideration of the 0.5 accuracy cut-off is primarily due to a strong linear correlation between the contact accuracy of the final models and the TM-scores of the models from C-QUARK, as shown in Fig. S19, where the PCCs are 0.904 and 0.877 for all- and long-range contacts, respectively. Such strong correlations indicate that selection of predicted contacts with an accuracy of at least 0.5 and the subsequent satisfaction of these contacts may lead to the generation of models with TM-scores [10] of at least 0.5, which is an indication of obtaining a similar fold as the native [11]. All the confidence cut-offs were determined based on the 243 training proteins (Dataset S1 in SI), which are non-homologous (with <30% sequence identity) to the 247 test proteins discussed in this work.

Text S3. C-QUARK force field used to guide the REMC simulations

In order to guide its REMC simulations, C-QUARK uses the following force field that calculates the total energy of a conformation by summing up 12 energy terms[12]:

$$E_{tot} = w_1 E_{prm} + w_2 E_{prs} + w_3 E_{ev} + w_4 E_{hb} + w_5 E_{sa} + w_6 E_{dh} + w_7 E_{dp} + w_8 E_{rg} + w_9 E_{bab} + w_{10} E_{hp} + w_{11} E_{c\alpha} + w_{12} E_{con} \tag{S2}$$

Here, the terms account for the backbone atomic pairwise potential (E_{prm}), side-chain center pairwise potential (E_{prs}), excluded volume (E_{ev}), hydrogen bonding (E_{hb}), solvent accessibility (E_{sa}), backbone torsion angles (E_{dh}), fragment-based distance profiles (E_{dp}), radius of gyration (E_{rg}), strand-helix-strand packing (E_{bab}), helix packing (E_{hp}), distance between adjacent Cα atoms ($E_{c\alpha}$), and the contact potential (E_{con}). While the first ten terms are used in both QUARK and C-QUARK, the final term, E_{con}, is unique to the C-QUARK force field and accounts for the contact restraints from the predicted contacts (see Eq. 1 and Fig. S17). In addition to the contact potential term, the 11th energy term, which factors in the distance between adjacent Cα atoms ($E_{c\alpha}$), is also a newly added term and takes the following form:

$$E_{c\alpha} = \sum_{i=1}^{L-1} I\left[d_{i,i+1} > 4\right]\left(d_{i,i+1} - 4\right)^2 \tag{S3}$$

where $d_{i,i+1}$ is the Cα-Cα distance between residues *i* and *i*+1, and *I*[ ] is the Iverson bracket, i.e., $I\left[d_{i,i+1} > 4\right]$=1 if $d_{i,i+1} > 4$, and 0 otherwise. This term is designed to penalize backbone breaks between adjacent residue pairs with Cα-Cα distances > 4Å, which can occur after fragment movements. All the weighting parameters in C-QUARK were re-tuned on the training protein set listed in Dataset S1, to appropriately balance the inherent force field with the contact restraints by maximizing the TM-score of the predicted models. As a result, most of the weighting parameters in w_{1-10} are similar to what was used in QUARK[12] despite the use of different training proteins, showing the robustness of the QUARK force field. It is interesting that the weight (w_7) of the distance-profile energy term increased from 0.60 to 3.00 in the C-QUARK force field to enlarge the effect of filtering out false positive contacts. The last parameter w_{12} is equal to 0.426 when N_f >50, and 0.355 otherwise.

Text S4. Well depth in the contact potential

The depth of the energy potential, U_{ij}, between residue pair *i* and *j* in the 3G contact potential is calculated as:

$$U_{ij} = \sum_{m=1}^{10} \left[2.5 * \left(1 + \left(\left(Cscore_{ij}\right)_m - \left(C_{0.5}^R\right)_m\right)\right)\right] \tag{S4}$$

where *m* is the number of contact predictors, $\left(Cscore_{ij}\right)_m$ is the confidence score of the predicted contact between residue pair *i* and *j* by the *m*th predictor, and $\left(C_{0.5}^R\right)_m$ is the confidence score cut-off listed in Table S17, which corresponds to an average accuracy =0.5 for the *m*th predictor at *R* ranges (short, medium and long) based on the training proteins.

SUPPLEMENTARY TABLES

Table S1: Average accuracies of all-range and long-range predicted contacts used in the C-QUARK simulations for *alpha*, *beta* and *alpha-beta* proteins. Additionally, the TM-score and success rate comparison between C-QUARK and QUARK on these proteins is presented, where the first models produced by the programs are considered for the comparison. The values in the parentheses of the fifth column show the p-values from one-sided Student's t-tests, indicating the significance of the TM-score comparison with respect to C-QUARK. The values in the parentheses of the sixth and seventh columns represent the percentage of the cases where the models obtained similar folds as the corresponding native structures.

Protein type	Contact prediction accuracy		Average TM-score		Number of cases with TM-score≥0.5	
	All-range	Long-range	C-QUARK	QUARK	C-QUARK	QUARK
Alpha (64)	0.398	0.377	0.636	0.502 (2.64×10^{-10})	52 (81%)	27 (42%)
Beta (67)	0.552	0.497	0.539	0.374 (2.84×10^{-13})	42 (63%)	15 (22%)
Alpha-beta (116)	0.548	0.520	0.629	0.408 (1.65×10^{-32})	92 (79%)	29 (25%)

Table S2: Average accuracies of the different ranges of predicted contacts used in the C-QUARK simulations. Additionally, the accuracies of the contacts derived from the first models of C-QUARK and QUARK are presented. The values in the parentheses show the p-values from one-sided Student's t-tests with respect to the predicted contacts used by C-QUARK.

Contact type	Short-range (6≤|i-j|<12)	Medium-range (12≤|i-j|<24)	Long-range (|i-j|≥24)	All-range (|i-j|≥6)
Predicted contacts used in C-QUARK	0.532	0.505	0.477	0.502
Contacts in C-QUARK models	0.551 (1.21×10^{-2})	0.537 (1.00×10^{-4})	0.482 (3.61×10^{-2})	0.516 (2.11×10^{-2})
Contacts in QUARK models	0.411 (4.42×10^{-12})	0.387 (1.47×10^{-15})	0.247 (1.46×10^{-37})	0.335 (1.16×10^{-30})

Table S3: Average satisfaction rates of different ranges of contacts in the first models generated by C-QUARK and QUARK based on the test set. The values in the parentheses represent the p-values calculated by one-sided Student's t-tests. The definition of contact satisfaction rate is discussed in Fig. S5.

Method	Short-range (6≤|i-j|<12)	Medium-range (12≤|i-j|<24)	Long-range (|i-j|≥24)	All-range (|i-j|≥6)
C-QUARK	0.564	0.551	0.509	0.537
QUARK	0.503 (1.18×10^{-18})	0.406 (3.09×10^{-44})	0.208 (9.03×10^{-74})	0.322 (2.01×10^{-74})

Table S4: Average TM-scores and GDT_TS scores (Global Distance Test Total Score) for the first models generated by C-QUARK, C-QUARK without the distance profile energy term, and C-QUARK without fragment-based optimization on the test set. The values in the parentheses of the second and third columns represent the p-values calculated by one-sided Student's t-tests. The values in parentheses in the fourth column represent the percentage of cases where the models obtained the same fold as the native. As per the CASP evaluation measurement, GDT_TS is calculated by GDT_TS = (GDT_P1 + GDT_P2 + GDT_P4 + GDT_P8)/4, where GDT_Pn denotes the percent of residues under the distance cut-off ⩽ n Å.

Method	Average TM-score	Average GDT_TS	Number of cases with TM-score≥0.5
C-QUARK	0.606	53.90	186 (75%)
C-QUARK (no distance profile term)	0.593 (4.16×10^{-4})	52.51 (1.70×10^{-5})	184 (74%)
C-QUARK (no fragments)	0.553 (1.59×10^{-30})	48.41 (2.79×10^{-33})	162 (66%)

Table S5: Average TM-scores, GDT_TS scores and RMSDs for the first models generated by C-QUARK, QUARK, CNS and DConStruct on the test set. The values in the parentheses of the second, third and fourth columns represent the p-values calculated by one-sided Student's t-tests. Additionally, the values in parentheses of the fifth column represent the percentage of the cases where the models obtained similar folds as the corresponding native structures.

Method	TM-score	GDT_TS	RMSD	Number of cases with TM-score≥0.5
C-QUARK	0.606	53.90	6.94	186 (75%)
QUARK	0.423 (6.80×10^{-51})	38.69 (2.08×10^{-36})	12.14 (1.8×10^{-29})	71 (29%)
CNS	0.530 (3.51×10^{-20})	46.51 (6.00×10^{-23})	8.47 (8.12×10^{-15})	143 (58%)
DConStruct	0.524 (1.76×10^{-25})	45.37 (5.62×10^{-28})	8.18 (3.11×10^{-12})	146 (59%)

Table S6: Average TM-scores, GDT_TS scores and RMSDs for the first models generated by C-QUARK, QUARK, CNS and DConStruct on the 59 targets of the test set with low contact-map prediction accuracy. The values in the parentheses of the second, third and fourth columns represent the p-values calculated by one-sided Student's t-tests. Additionally, the values in parentheses of the fifth column represent the percentage of the cases where the models obtained similar folds as the corresponding native structures.

Method	TM-score	GDT_TS	RMSD	Number of cases with TM-score≥0.5
C-QUARK	0.428	39.98	10.21	24 (41%)
QUARK	0.348 (1.36×10^{-6})	33.37 (6.53×10^{-6})	14.15 (1.13×10^{-6})	7 (12%)
CNS	0.324 (1.52×10^{-9})	30.25 (1.66×10^{-9})	12.65 (4.06×10^{-7})	4 (7%)
DConStruct	0.326 (3.02×10^{-9})	30.14 (3.46×10^{-9})	12.16 (6.59×10^{-5})	4 (7%)

Table S7: Average TM-scores, GDT_TS scores and RMSDs for the first models generated by C-QUARK, QUARK, CNS, DConStruct and trRosetta on the 57 targets of the test set without redundancy to the trRosetta training set and all training sets of the contact predictors used by C-QUARK. Here, trRosetta used only the contact restraints, i.e., distances where the peak of the predicted distance distribution was lower than 8Å or the sum of probabilities below 8Å was greater than 0.5, to provide a fair comparison with C-QUARK. The values in the parentheses of the second, third and fourth columns represent the p-values based on one-sided Student's t-tests. Additionally, the values in parentheses of the fifth column represent the percentage of the cases where the models obtained similar folds as the corresponding native structures.

Method	TM-score	GDT_TS	RMSD	Number of cases with TM-score≥0.5
C-QUARK	0.525	48.19	8.64	30 (53%)
QUARK	0.418 (8.63×10^{-7})	39.19 (3.84×10^{-6})	13.09 (6.28×10^{-7})	17 (30%)
CNS	0.440 (2.61×10^{-9})	39.70 (4.34×10^{-9})	10.48 (2.70×10^{-6})	21 (37%)
DConStruct	0.438 (3.64×10^{-9})	39.07 (2.38×10^{-9})	9.95 (6.83×10^{-4})	21 (37%)
trRosetta (contact)	0.463 (3.96×10^{-2})	42.31 (5.43×10^{-2})	10.47 (4.91×10^{-3})	20 (35%)

Table S8: The average TM-scores and GDT_TS scores of the first models by C-QUARK on the CASP targets in comparison to the top five servers on FM, FM/TBM and TBM-hard targets in CASP13. The values in the parentheses are the p-values calculated by one-sided Student's t-tests between C-QUARK and the other control programs. We did not show 'Zhang-Server' in CASP13 because it used C-QUARK models as the starting models for FM targets.

Target type	Methods	Average TM-score	Average GDT_TS
All (64 targets)	C-QUARK (participated as "QUARK")	0.588	52.09
	RaptorX-DeepModeller	0.558 (2.24×10^{-2})	49.38 (1.89×10^{-2})
	RaptorX-Contact	0.531 (3.31×10^{-4})	46.56 (8.90×10^{-5})
	RaptorX-TBM	0.521 (1.94×10^{-6})	45.92 (2.99×10^{-6})
	BAKER-ROSETTASERVER	0.513 (2.47×10^{-4})	45.76 (6.86×10^{-4})
	Zhou-SPOT-3D	0.447 (1.15×10^{-9})	38.77 (7.61×10^{-10})
TBM-hard (21 targets)	C-QUARK (participated as "QUARK")	0.720	61.03
	RaptorX-DeepModeller	0.682 (7.35×10^{-2})	58.04 (3.58×10^{-2})
	RaptorX-Contact	0.613 (5.88×10^{-4})	50.97 (3.60×10^{-4})
	RaptorX-TBM	0.686 (8.39×10^{-2})	58.11 (3.55×10^{-2})
	BAKER-ROSETTASERVER	0.644 (1.96×10^{-1})	54.69 (2.47×10^{-1})
	Zhou-SPOT-3D	0.576 (3.04×10^{-4})	46.40 (1.09×10^{-3})
FM/TBM (12 targets)	C-QUARK (participated as "QUARK")	0.598	58.94
	RaptorX-DeepModeller	0.572 (3.39×10^{-1})	56.45 (1.79×10^{-1})
	RaptorX-Contact	0.525 (4.47×10^{-2})	51.54 (1.78×10^{-2})
	RaptorX-TBM	0.538 (1.05×10^{-2})	53.21 (2.89×10^{-2})
	BAKER-ROSETTASERVER	0.609 (6.54×10^{-1})	60.58 (7.01×10^{-1})
	Zhou-SPOT-3D	0.489 (3.36×10^{-2})	48.91 (2.88×10^{-2})
FM (31 targets)	C-QUARK (participated as "QUARK")	0.495	43.38
	RaptorX-DeepModeller	0.468 (1.32×10^{-1})	40.79 (9.62×10^{-2})
	RaptorX-Contact	0.477 (1.60×10^{-1})	41.64 (1.51×10^{-1})
	RaptorX-TBM	0.402 (1.23×10^{-4})	34.84 (1.24×10^{-4})
	BAKER-ROSETTASERVER	0.388 (5.92×10^{-5})	33.98 (8.85×10^{-5})
	Zhou-SPOT-3D	0.343 (7.87×10^{-8})	29.68 (1.07×10^{-7})

Table S9: The average TM-scores and GDT_TS scores of the first models produced by C-QUARK, AlphaFold and trRosetta on the CASP13 targets in the FM, FM/TBM and TBM-hard categories. The values in the parentheses are the p-values calculated by one-sided Student's t-tests between C-QUARK and the other control programs.

Target type	Methods	Average TM-score	Average GDT_TS
All (64 targets)	C-QUARK	0.588	52.09
	AlphaFold	0.648 ($1.00 \times 10^{+0}$)	58.43 ($1.00 \times 10^{+0}$)
	trRosetta	0.619 (9.99×10^{-1})	55.34 (9.97×10^{-1})
TBM-hard (21 targets)	C-QUARK	0.720	61.03
	AlphaFold	0.710 (5.41×10^{-1})	61.80 (5.90×10^{-1})
	trRosetta	0.680 (1.92×10^{-1})	57.93 (1.64×10^{-1})
FM/TBM (12 targets)	C-QUARK	0.598	58.94
	AlphaFold	0.695 (9.98×10^{-1})	68.22 (9.89×10^{-1})
	trRosetta	0.622 (7.50×10^{-1})	61.56 (8.03×10^{-1})
FM (31 targets)	C-QUARK	0.495	43.38
	AlphaFold	0.589 ($1.00 \times 10^{+0}$)	52.35 ($1.00 \times 10^{+0}$)
	trRosetta	0.577 ($1.00 \times 10^{+0}$)	51.17 ($1.00 \times 10^{+0}$)

Table S10: Performance of C-QUARK on 21 CASP13 multi-domain proteins. The "Target" column is the name of each target. The second and third columns are the number of domains and the domain boundaries given by the CASP13 assessors for each target. The fourth column shows the TM-score of the C-QUARK first models for the full-length targets, and the fifth column shows the TM-scores of the C-QUARK first model for each individual domain of the targets. The last column is the average TM-score of the individual domains for each target.

Target	No. of domains	domain	TM-score of full-length model	TM-scores of domain models	Average TM-score of domain models
T0953s2	3	2-45;46-151,229-249; 152-228;	0.459	0.361,0.465, 0.286	0.371
T0957s1	2	2-37,92-163;38-91;	0.385	0.397,0.378	0.388
T0960	5	11-42;43-126; 127-215;216-279; 280-384;	0.289	0.158,0.437, 0.765,0.236, 0.714	0.462
T0963	5	9-39;40-121; 122-214;215-278; 279-372;	0.251	0.160,0.459, 0.773,0.250, 0.786	0.486
T0976	2	9-128;129-252;	0.703	0.839,0.830	0.835
T0977	2	59-359;360-563;	0.630	0.899,0.777	0.838
T0981	5	34-119;120-190,394-402; 191-393;403-513; 514-640;	0.317	0.535,0.251, 0.669,0.591, 0.640	0.537
T0982	2	11-145;146-277;	0.484	0.867,0.590	0.729
T0984	2	39-406,565-700;417-563;	0.863	0.875,0.789	0.832
T0987	2	11-195;196-402;	0.380	0.581,0.438	0.510
T0989	2	1-134;135-246;	0.364	0.477,0.313	0.395
T0990	3	1-76;77-134,348-520; 135-347;	0.241	0.577,0.371, 0.223	0.390
T0996	6	17-123;124-250; 251-350;351-483; 484-604;605-708;	0.350	0.779,0.820, 0.816,0.750, 0.737,0.865	0.795
T0999	5	15-400;401-853; 866-1045;1046-1289; 1290-1577;	0.429	0.986,0.769, 0.792,0.964, 0.890	0.880
T1000	2	10-92;93-523;	0.711	0.9480,0.851	0.900
T1002	3	1-59;60-118; 127-270;	0.469	0.782,0.802, 0.777	0.787
T1004	3	66-151;152-228; 229-458;	0.558	0.793,0.673, 0.926	0.797
T1011	2	55-268,433-520;271-430;	0.580	0.792,0.874	0.833
T1014	2	1-159;160-276;	0.528	0.897,0.800	0.849
T1021s3	2	4-181;195-295;	0.426	0.636,0.452	0.544
T1022s1	2	2-157;158-224;	0.435	0.555,0.632	0.594

Table S11: Top L contact prediction accuracy for different predictors and consensus from the predictors at different ranges based on the test proteins listed in Dataset S2, where L is the length of the proteins. The predictors are categorized based on the long-range accuracy. Here, accuracies are defined as $ACC = \frac{TP}{TP+FP}$, where TP and FP are true and false positive predictions among the top L predictions.

Category	Predictors	Short-range (6≤|i-j|<12)	Medium-range (12≤|i-j|<24)	Long-range (|i-j|≥24)	All-range (|i-j|≥6)
Very high	ResPRE	0.286	0.357	0.538	0.724
	NeBcon	0.283	0.356	0.539	0.666
	DeepPLM	0.282	0.347	0.518	0.696
High	DNCON2	0.283	0.344	0.494	0.680
	Deepcontact	0.258	0.333	0.475	0.642
	DeepCov	0269	0.324	0.457	0.643
Medium	MetaPSICOV2	0267	0.311	0.424	0.600
Low	GREMLIN	0.171	0.204	0.291	0.395
	CCMpred	0.171	0.205	0.290	0.394
	FreeContact	0.144	0.172	0.254	0.336
	Combined	0.532	0.510	0.561	0.722

Table S12: Top $L/2$ contact prediction accuracy for different predictors and consensus from the predictors at different ranges based on the test proteins listed in Dataset S2, where L is the length of the proteins. The predictors are categorized based on the long-range accuracy. Here, accuracies are defined as $ACC = \frac{TP}{TP+FP}$, where TP and FP are true and false positive predictions among the top $L/2$ predictions.

Category	Predictors	Short-range (6≤|i-j|<12)	Medium-range (12≤|i-j|<24)	Long-range (|i-j|≥24)	All-range (|i-j|≥6)
Very high	ResPRE	0.493	0.552	0.685	0.828
	NeBcon	0.485	0.549	0.674	0.766
	DeepPLM	0.478	0.533	0.660	0.797
High	DNCON2	0.477	0.520	0.635	0.789
	Deepcontact	0.416	0.504	0.617	0.755
	DeepCov	0.446	0.489	0.602	0.758
Medium	MetaPSICOV2	0.421	0.465	0.552	0.704
Low	Gremlin	0.230	0.274	0.404	0.499
	CCMpred	0.230	0.277	0.402	0.498
	FreeContact	0.209	0.257	0.346	0.419
	Combined	0.558	0.581	0.692	0.824

Table S13: Top L contact prediction accuracy for different predictors at different ranges based on the training proteins listed in Dataset S1, where L is the length of the proteins. The predictors are categorized based on the long-range accuracy. Here, accuracies are defined as $ACC = \frac{TP}{TP+FP}$, where TP and FP are true and false positive predictions among the top L predictions.

Category	Predictors	Short-range (6≤|i-j|<12)	Medium-range (12≤|i-j|<24)	Long-range (|i-j|≥24)	All-range (|i-j|≥6)
Very high	ResPRE	0.291	0.360	0.578	0.764
	NeBcon	0.285	0.355	0.575	0.697
	DeepPLM	0.285	0.369	0.550	0.740
High	DNCON2	0.286	0.341	0.522	0.702
	Deepcontact	0.267	0.342	0.499	0.671
	DeepCov	0.275	0.334	0.474	0.675
Medium	MetaPSICOV2	0265	0.320	0.443	0.627
Low	GREMLIN	0.171	0.199	0.294	0.407
	CCMpred	0.174	0.203	0.301	0.415
	FreeContact	0.148	0.178	0.267	0.348

Table S14: Top $L/2$ contact prediction accuracy for different predictors at different ranges based on the training proteins listed in Dataset S1, where L is the length of the proteins. The predictors are categorized based on the long-range accuracy. Here, accuracies are defined as $ACC = \frac{TP}{TP+FP}$, where TP and FP are true and false positive predictions among the top $L/2$ predictions.

Category	Predictors	Short-range (6≤|i-j|<12)	Medium-range (12≤|i-j|<24)	Long-range (|i-j|≥24)	All-range (|i-j|≥6)
Very high	ResPRE	0.504	0.566	0.732	0.865
	NeBcon	0.491	0.556	0.716	0.798
	DeepPLM	0.489	0.564	0.703	0.842
High	DNCON2	0.485	0.530	0.667	0.809
	Deepcontact	0.433	0.519	0.646	0.782
	DeepCov	0.460	0.511	0.626	0.792
Medium	MetaPSICOV2	0.430	0.483	0.575	0.739
Low	Gremlin	0.233	0.286	0.417	0.527
	CCMpred	0.238	0.294	0.424	0.538
	FreeContact	0.213	0.262	0.366	0.441

Table S15: Selection of width of the first well (d_b) in the contact potential at various lengths (L) of proteins based on the 243 training proteins (Dataset S1).

	L<100	L>=100 & L<120	L >= 120 & L<200	L>200
Start of 1st well	8	8	8	8
Width of 1st well, d_b	6	8	10	12
End of 1st & start of 2nd well, $D=(8+d_b)$	14	16	18	20
Width of 2nd well, (80-D)	66	64	62	60

Table S16: Selection of the least number of contacts as restraints in the folding simulations from different contact predictors at various lengths (*L*), and effective number of sequences (N_f) available in the multiple sequence alignments (MSAs). The definition of N_f is discussed in Text S1.

Category	Predictors	N_f <50	N_f ≥ 50 & *L*<120	N_f ≥ 50 & *L*≥120
Very high	ResPRE	*L*	*L/2*	*L/1.25*
	NeBcon	*L*	*L/2*	*L/1.25*
	DeepPLM	*L*	*L/2*	*L/1.25*
High	DNCON2	*L/2*	*L/3*	*L/2.25*
	Deepcontact	*L/2*	*L/3*	*L/2.25*
	DeepCov	*L/2*	*L/3*	*L/2.25*
Medium	MetaPSICOV2	*L/4.5*	*L/5.5*	*L/4.75*
Low	GREMLIN	*L/7.5*	*L/5.5*	*L/7.75*
	CCMpred	*L/7.5*	*L/5.5*	*L/7.75*
	FreeContact	*L/7.5*	*L/5.5*	*L/7.75*

Table S17: Confidence score cut-offs of different contact predictors that correspond to an accuracy of 0.5 based on the 243 training proteins (Dataset S1). Definition of the accuracy at different confidence score is discussed in Fig. S4.

Category	Predictors	All-range	Short-range	Medium-range	Long-rangee
Very high	ResPRE	0.626	0.607	0.581	0.654
	NeBcon	0.801	0.483	0.626	0.849
	DeepPLM	0.965	0.962	0.959	0.968
High	DNCON2	0.474	0.482	0.484	0.457
	Deepcontact	0.983	0.977	0.983	0.987
	DeepCov	0.571	0.551	0.546	0.592
Medium	MetaPSICOV2	0.606	0.588	0.638	0.597
Low	GREMLIN	0.681	0.753	0.678	0.656
	CCMpred	0.676	0.769	0.666	0.644
	FreeContact	0.844	0.921	0.756	0.819

SUPPLEMENTARY FIGURES

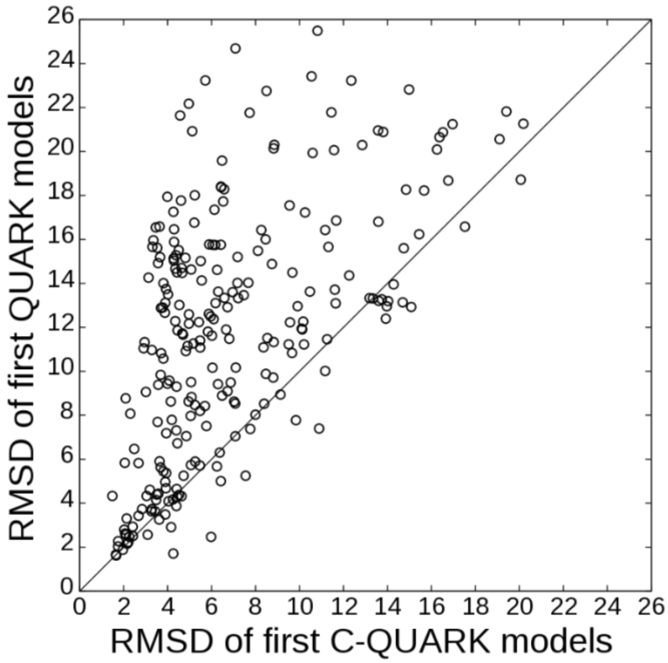

Figure S1: RMSD comparison between the first models produced by C-QUARK and QUARK based on the 247 test proteins. Points above the diagonal line indicate models with better quality produced by C-QUARK than QUARK, and vice versa.

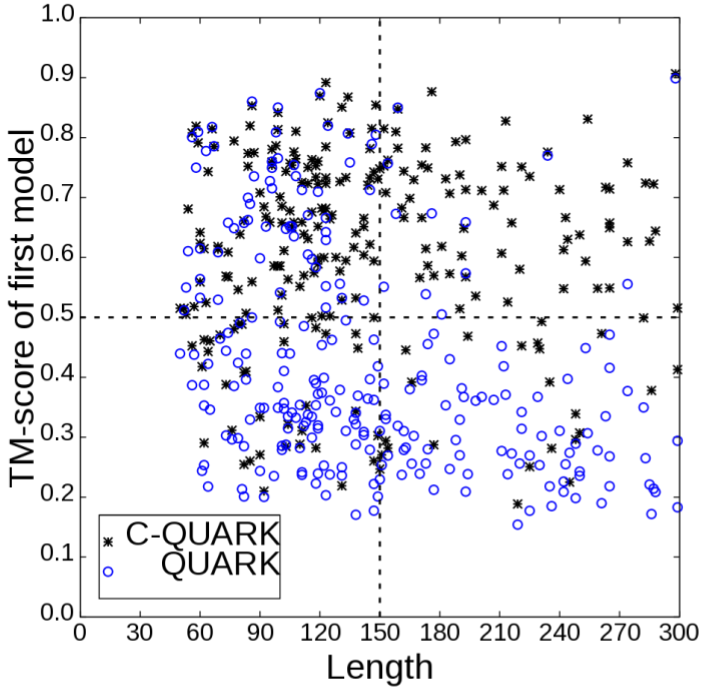

Figure S2: A scatter plot illustrating the TM-scores of the models produced by C-QUARK and QUARK for all the proteins at different lengths.

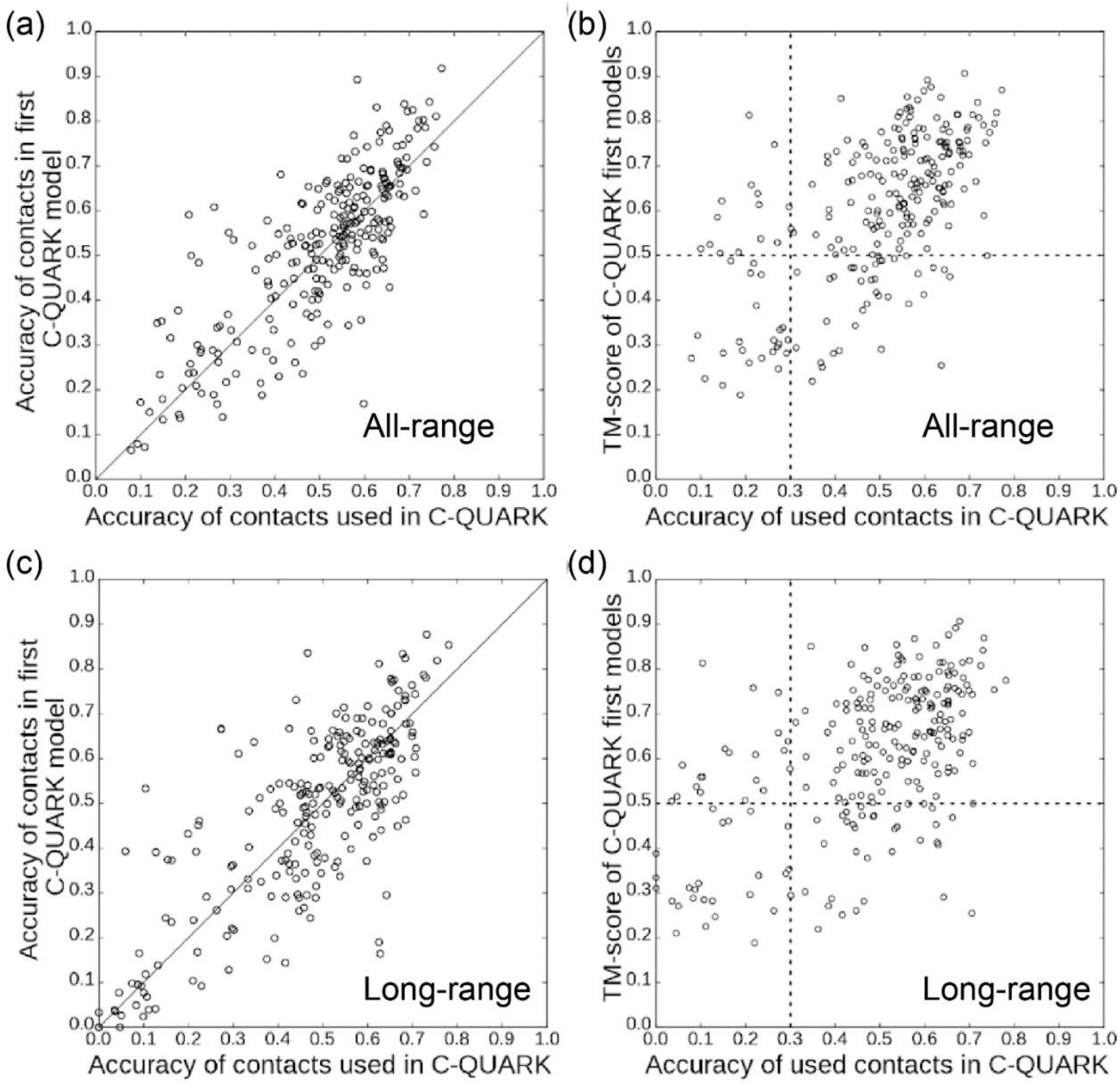

Figure S3: Accuracy of the predicted (A) all-range and (C) long-range contacts used in C-QUARK versus accuracy of the all-range and long-range contacts in the first models of C-QUARK, respectively. Accuracy of the predicted (B) all-range and (D) long-range contacts versus TM-scores of the first C-QUARK models. The horizontal dashed lines indicate the TM-score cut-off of 0.5, beyond which models are considered to obtain similar folds as the corresponding native structures. The vertical dashed lines indicate the contact prediction accuracy of 0.30, which is very low. Out of 38 targets in the test set that have all-range prediction accuracy < 0.30, C-QUARK generated models with TM-score>=0.5 for 14 of them (i.e. 37% of the cases).

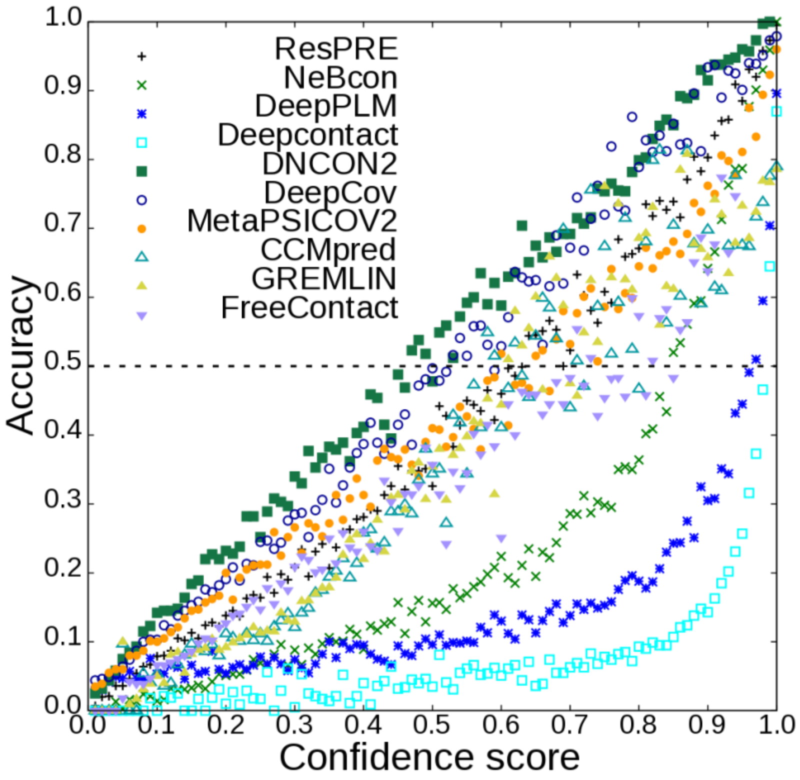

Figure S4: Confidence scores vs. corresponding long-range accuracies for each contact predictor. Here, we split the confidence score into 100 bins, where the bin width is 0.01. The accuracy for each bin of the confidence score ($cscore_i$) is calculated using the following equation:

$$accuracy\ (cscore_i) = \frac{N_{true}(cscore_i)}{N_{total}(cscore_i)}$$

Here, $N_{true}(cscore_i)$= number of true predicted contacts in the ith bin of $cscore$, and $N_{total}(cscore_i)$ = total number of predicted contacts in the ith bin of $cscore$. The dashed line corresponds to an accuracy of 0.5.

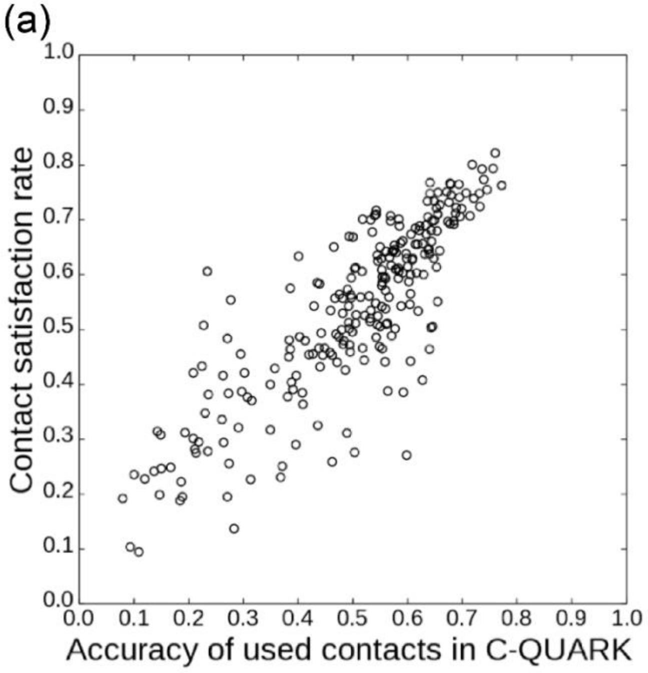

Figure S5: (A) Accuracy of the predicted contacts used in C-QUARK versus satisfaction rate of these contacts in the first models is shown. Here, the accuracy is based on long-range contacts. The PCC is 0.796 for the long-range contacts, indicating strong correlation between contact satisfaction and the accuracy of the contacts. While plots are not shown here, the PCCs for short-, medium- and all-ranges are 0.847, 0.798 and 0.842, respectively. (B) Dependence of satisfaction rates of different ranges of contacts in the first models on the number of predicted contacts used in the C-QUARK simulations. Contact satisfaction rate (*M*) for different ranges (*R*) is calculated using the following equation:

$$M(R) = \frac{N_{satisfied}\ (R)}{N_{total}\ (R)}$$

Here, $N_{total}\ (R)$= Total number of *R*-range contacts used during the C-QUARK simulations, and $N_{satisfied}\ (R)$= Total number of *R*-range contacts satisfied in the final models, where *R* refers to short-, medium-, long- and all-range.

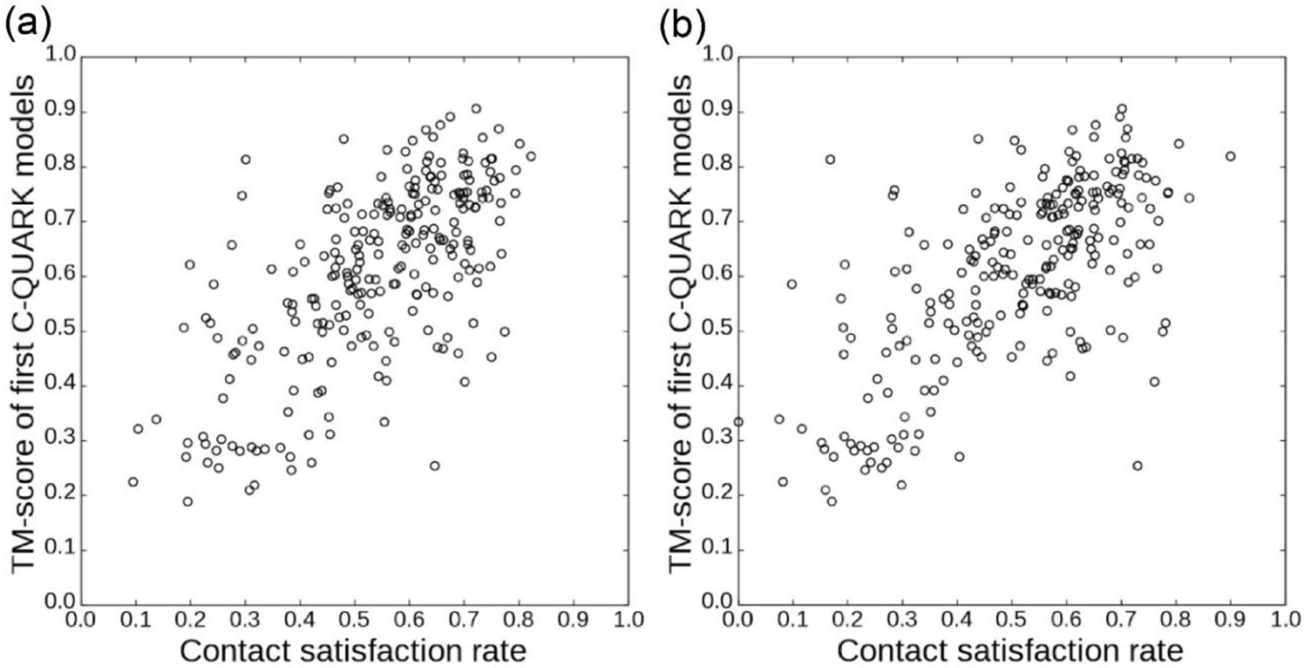

Figure S6: Effect of satisfaction rate of (A) all-range and (B) long-rage contacts on the TM-score of the first C-QUARK model. The PCCs are 0.665 and 0.672, respectively, for all- and long-range contacts.

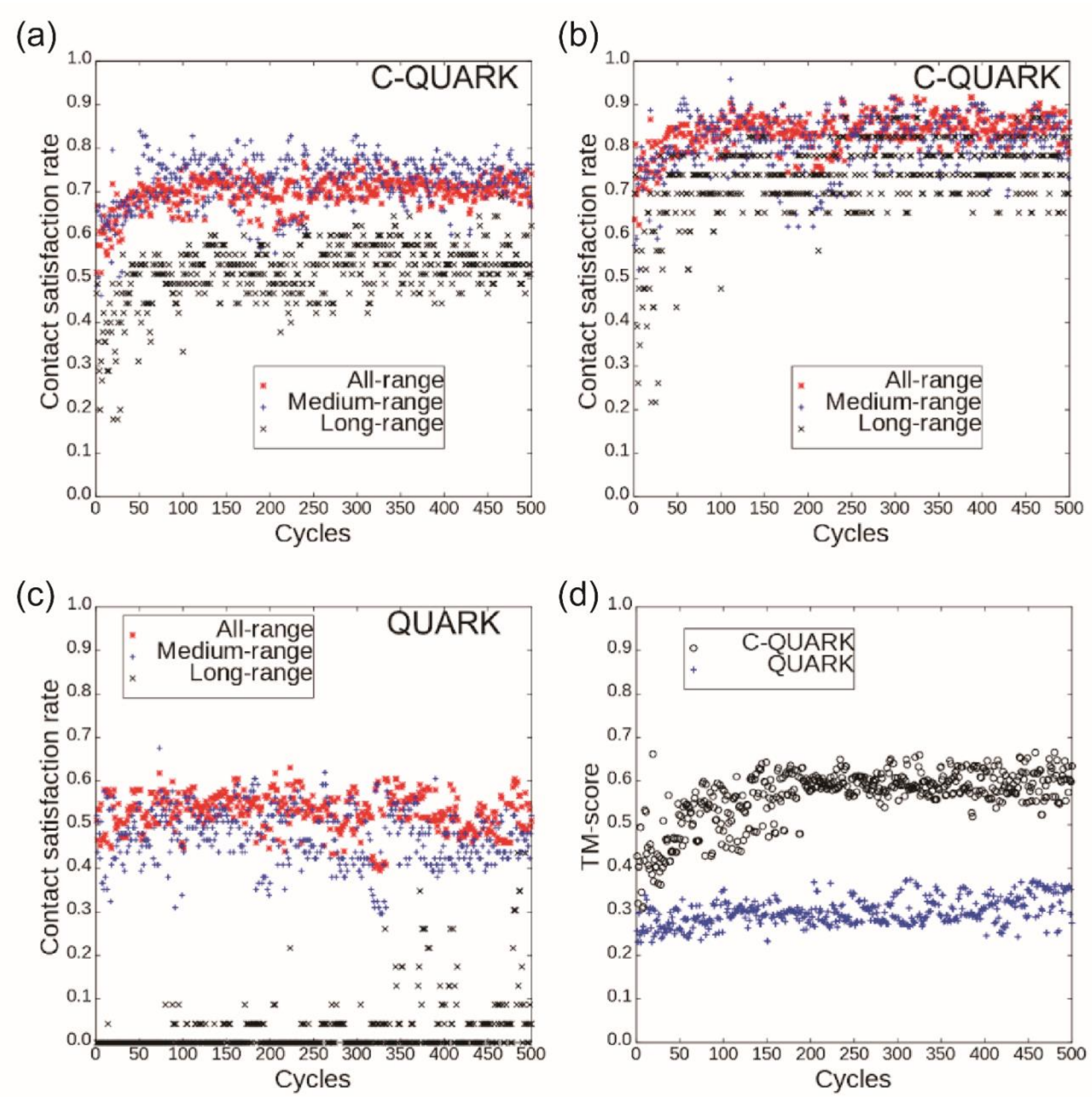

Figure S7: A representative case, 1jiwI, to demonstrate the increase in the contact satisfaction rate as the simulation cycles progress during a representative replica from the folding simulations. Satisfaction rates of (A) all contacts and (B) top 1.5*L* contacts used during the C-QUARK simulations are shown, where satisfaction rates are higher for the latter case. (C) Satisfaction rate of the top 1.5*L* predicted contacts in the QUARK simulation, where the satisfaction rate of contacts, particularly long-range contacts, is significantly lower compared to that of C-QUARK due to the lack of contact restraints and a contact potential in QUARK. (D) TM-score comparison of the decoys produced during the representative replica of the C-QUARK and QUARK simulations as the cycles progress. The increase in TM-score as the number of cycles increases is partly due to the satisfaction of predicted contacts in C-QUARK.

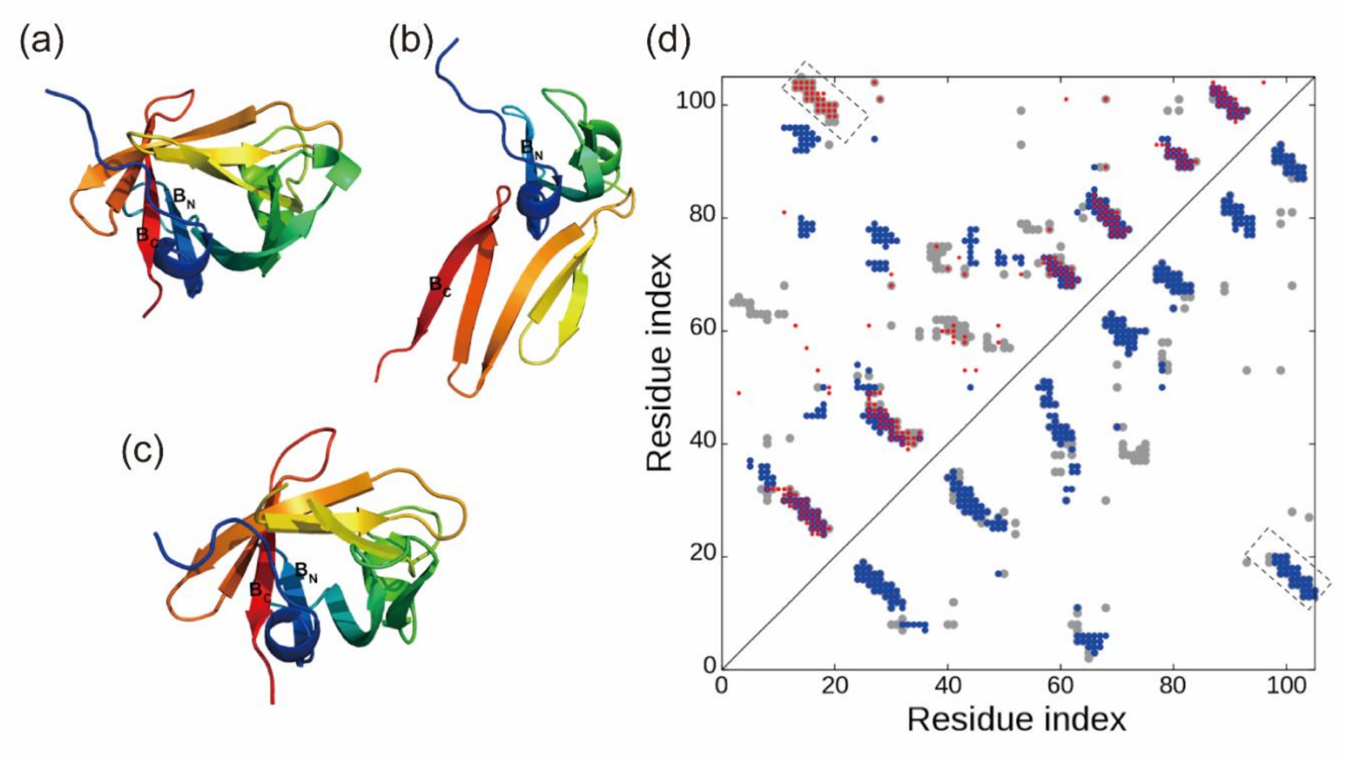

Figure S8: A similar representative case as Fig. S7 from 1jiwI, which illustrates how contact restraints help bring the contacting residues close to each other in 3D space as the folding simulations progress. (A), (B) and (C) represent the structure of the native, and decoys at an initial stage and at the final stage, respectively. (D) The contact-maps derived from the structures of the native protein (grey circle), and the decoys at the initial stage (blue circle in upper left triangle), and at the final stage (blue circle in lower right triangle). Additionally, the red circles in the left triangles represent the predicted contacts used as restraints in the C-QUARK simulations. All structures are colored in spectrum, with blue to red indicating the N- to C- terminal regions. B_N and B_C represent the beta-strands at the N- and C-termini, respectively. B_N and B_C are ~13.5 Å apart from each other after the first cycle of the simulation, as shown in (B), while these should be in contact (~4.4 Å) as evident from the native structure and in the contact-maps in (D) (highlighted with a rectangle in the upper left triangle). Restraints from the predicted contacts between the beta-strands help to bring these beta-strands close to each other at a distance of ~4.5 Å after 500 cycles, as shown in (C) and highlighted with a rectangle in the lower triangle of (D).

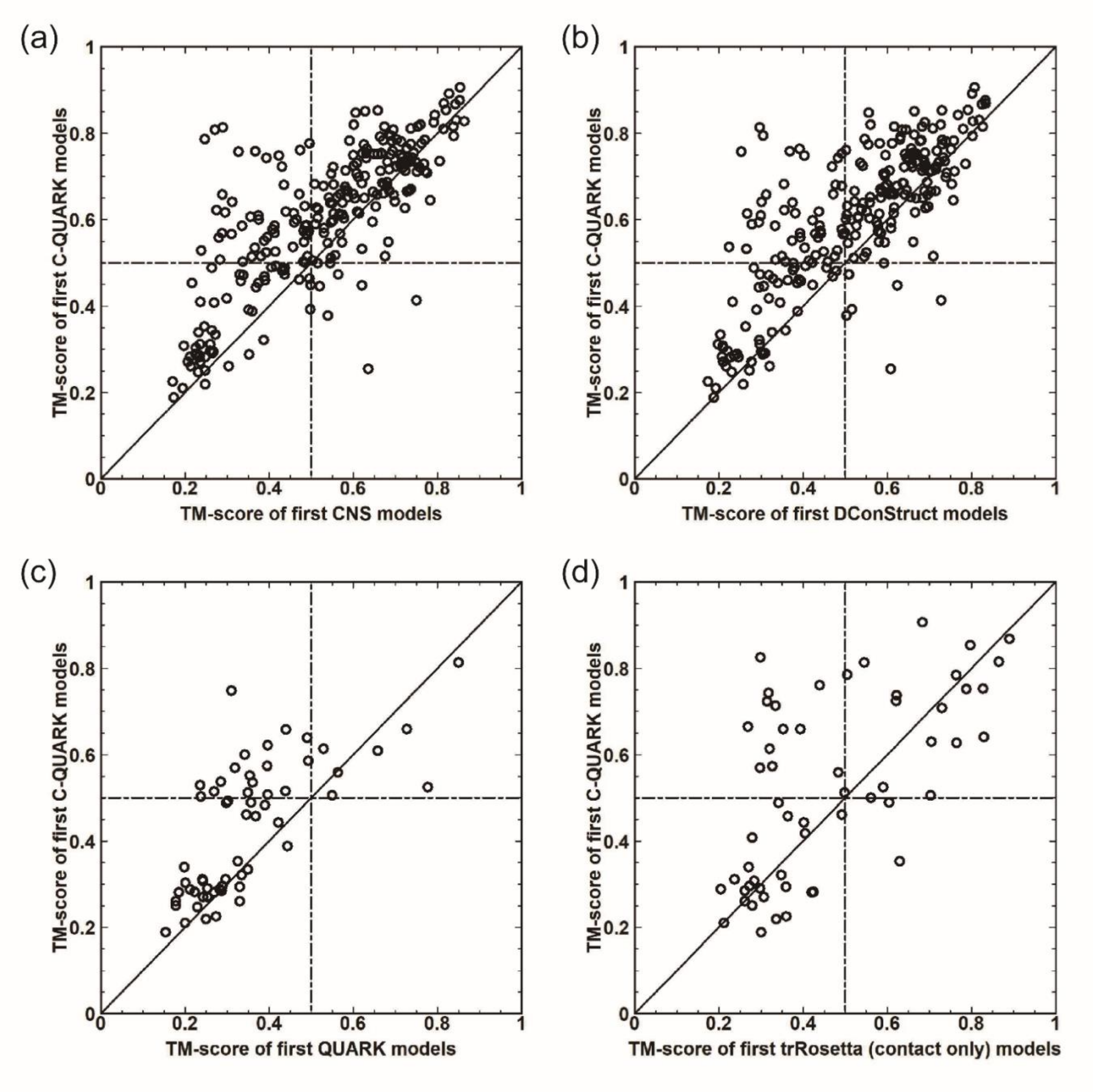

Figure S9: TM-score comparison between the first models produced by C-QUARK and CNS (A), and C-QUARK and DConStruct (B) for the 247 test proteins. TM-score comparison between the first models produced by C-QUARK and QUARK (C) for the 59 test proteins with low accuracy contact-map prediction. TM-score comparison between the first of C-QUARK and trRosetta (D) for 57 test proteins without redundancy to training sets of trRosetta and all contact predictors used in C-QUARK. The dashed lines indicate the TM-score cut-off of 0.5, beyond which models are considered to obtain similar folds as the corresponding native structures. Points above the diagonal line indicate models with better quality by C-QUARK than the control methods, and vice versa.

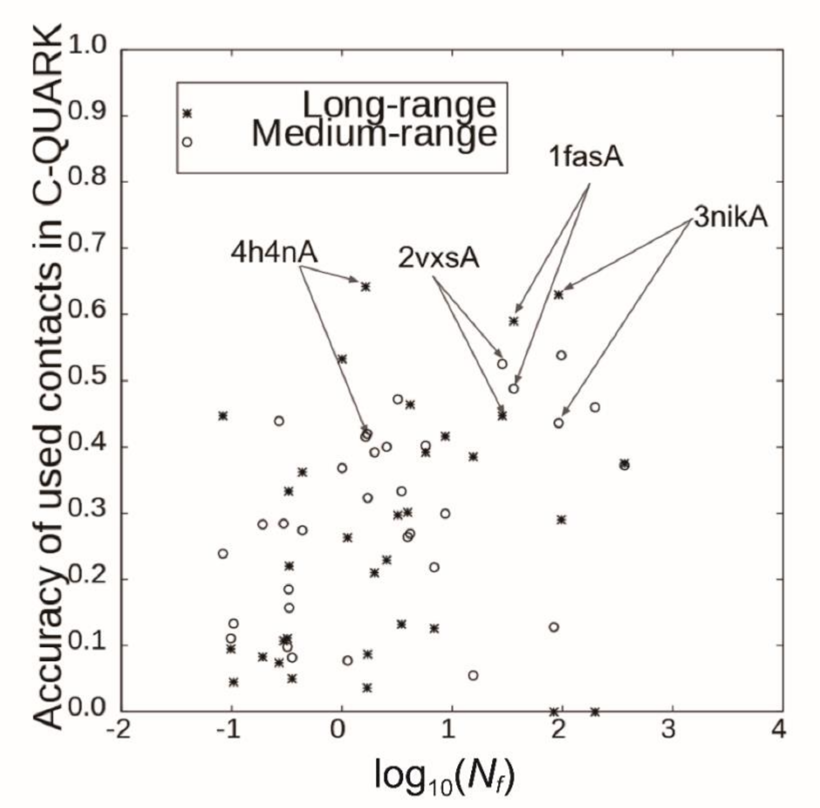

Figure S10: Accuracy of long-range and medium-range predicted contacts used in C-QUARK versus N_f for the cases that were not folded by C-QUARK. The majority of the proteins were not folded due to low contact prediction accuracy. However, four targets: 1fasA, 2vxsA, 3nikA and 4h4nA, as highlighted in the plot, have reasonable accuracies (>0.4) for the medium- and long-range predicted contacts, but are still not foldable by C-QUARK due to incorrect secondary structure prediction, and lack of predicted contacts in loop/coil regions, as demonstrated in the main text and Fig. S11.

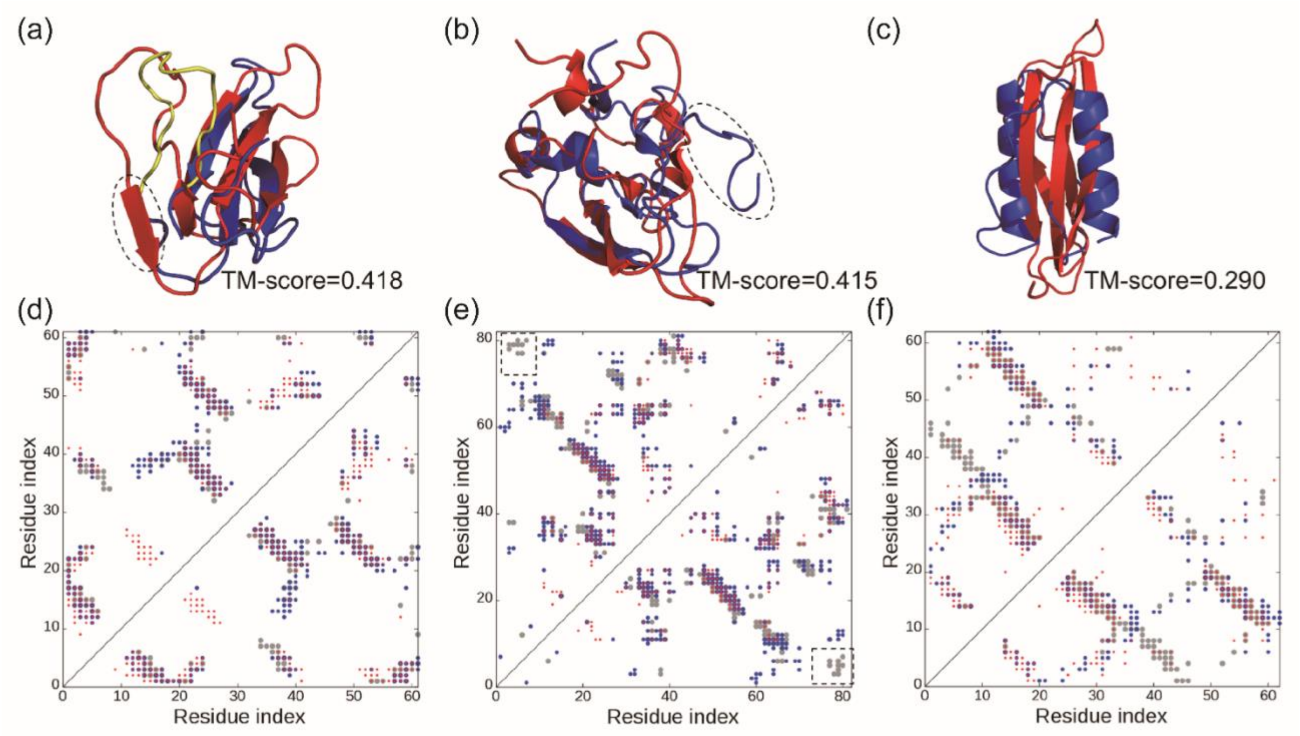

Figure S11: Example cases that are not folded by C-QUARK although the long-range and medium-range contact prediction accuracy for these proteins are reasonable (>0.4). (A), (B), (C) show the structures of native (red) and best in top five C-QUARK models (blue) for 1fasA, 3nikA and 4h4nA, respectively. (D), (E) and (F) represent the corresponding contact-maps of these proteins, respectively, for the native structure (grey circles), C-QUARK model (blue) and predicted (red) by the contact predictors. Here, contact-maps in the upper left triangles are similar to that in the lower right triangles in each contact plot. There is a mis-classification of the secondary structure predictor at the positions of 14-16 in 1fasA, where the region is classified as a coil instead of a beta-strand, as highlighted with a dashed circle in (A). As a result, the region from first position to position 16 is modeled as a coil instead of a beta sheet, where the yellow region, as highlighted in (A) is modeled as a floppy coil instead of a regular beta strand. In 3nikA, there is a lack of prediction of contacts in the coil region at the N-terminal, while there are supposed to be long range contacts between the residues at N- and that at C-terminal, as observed in the native structure and the corresponding contact-map, highlighted within dashed rectangle in (E). Consequently, the coil region at the N-terminal is not correctly modeled by the C-QUARK model, as highlighted with a dashed circle in (B). In the 4h4nA, while the regions at 27-35 and 51-58 are supposed to be beta-strands, the secondary structure predictor predicted these regions as helices. As a result, C-QUARK generates those models as helices, as shown in in (C).

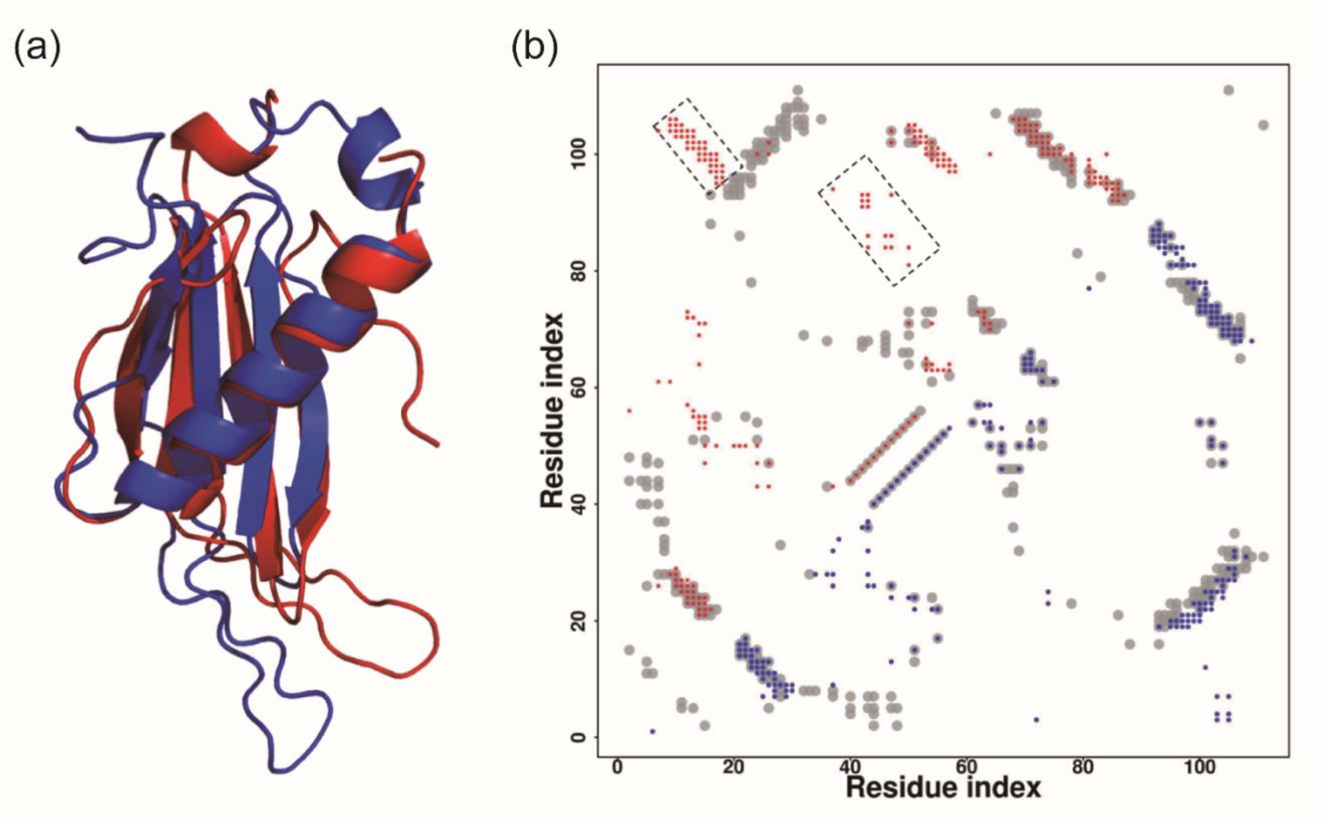

Figure S12: Case study for T0980s1-D1. (A) Superimposed structure of the C-QUARK first model (blue) on the native structure (red) of the FM target, T0980s1-D1, released in CASP13. The TM-score of the model is 0.540, indicating the model has a similar fold as the native. (B) The contact-maps extracted from the native structure (grey circle) and C-QUARK model (blue circles in the lower right triangle), and predicted contacts (red circles in upper left triangle) are shown. The rectangles in (B) highlight the falsely predicted contacts, which are not satisfied in the model due to other energy terms used during the C-QUARK simulations.

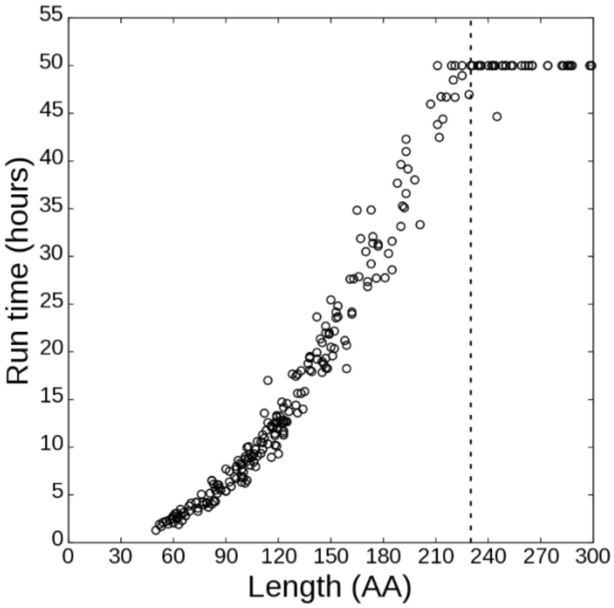

Figure S13: Dependence of C-QUARK run time on protein length. Dependence of the REMC simulation run time by C-QUARK and the length of the proteins from the test set. The simulations were terminated after 50 hours. The dashed line indicates the length (~230AA) beyond which the simulations were terminated and hence 500 cycles were not completed.

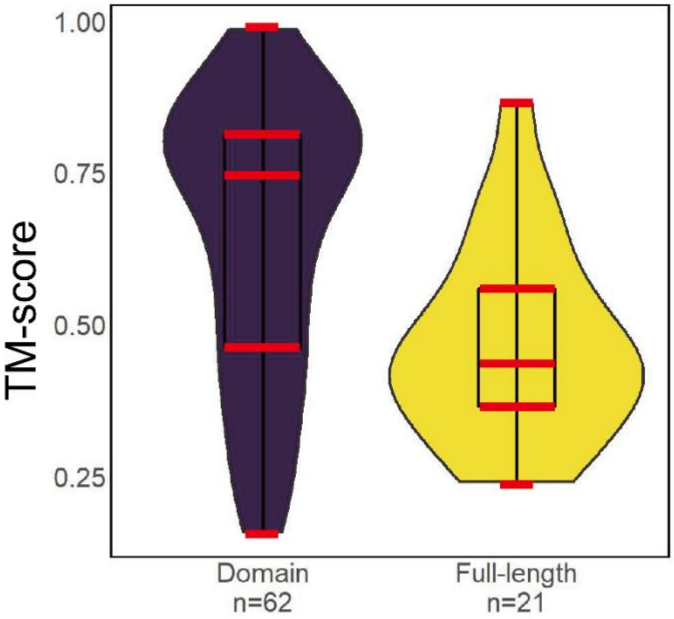

Figure S14: Boxplot and distribution of TM-scores for the first models produced by C-QUARK on 21 CASP13 multi-domain targets (yellow) and the corresponding 62 individual domains (purple). The 62 domains were generated from the 21 full-length multi-domain targets, where the domain boundaries were taken from the CASP official definitions. Here, all 21 multi-domain targets with solved experimental structures were selected for comparison, no specific target was removed from the CASP13 released target list. The horizontal axis indicates the counts of TM-scores and the vertical axis is TM-score of predicted models, where the “minimum”, “first quartile (Q1/25th Percentile)”, “median (Q2/50th Percentile)”, “third quartile (Q3/ 75th Percentile)”, and “maximum” of each boxplot are shown by bold red lines accordingly.

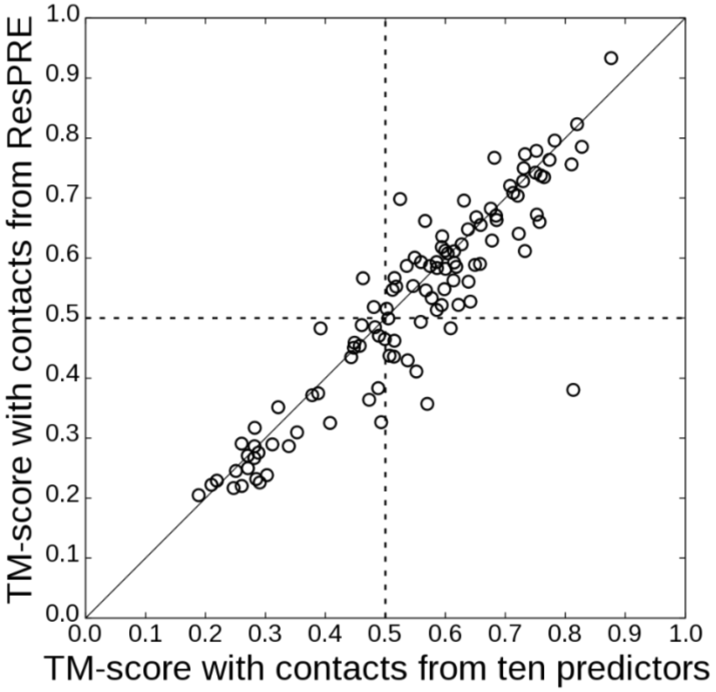

Figure S15: TM-score comparison of C-QUARK models produced based on contacts from all ten predictors and those from ResPRE, the contact predictor with the highest prediction accuracy, on 109 hard targets. The dashed lines indicate the TM-score cut-off of 0.50, beyond which models are considered to obtain similar folds as the corresponding native structures.

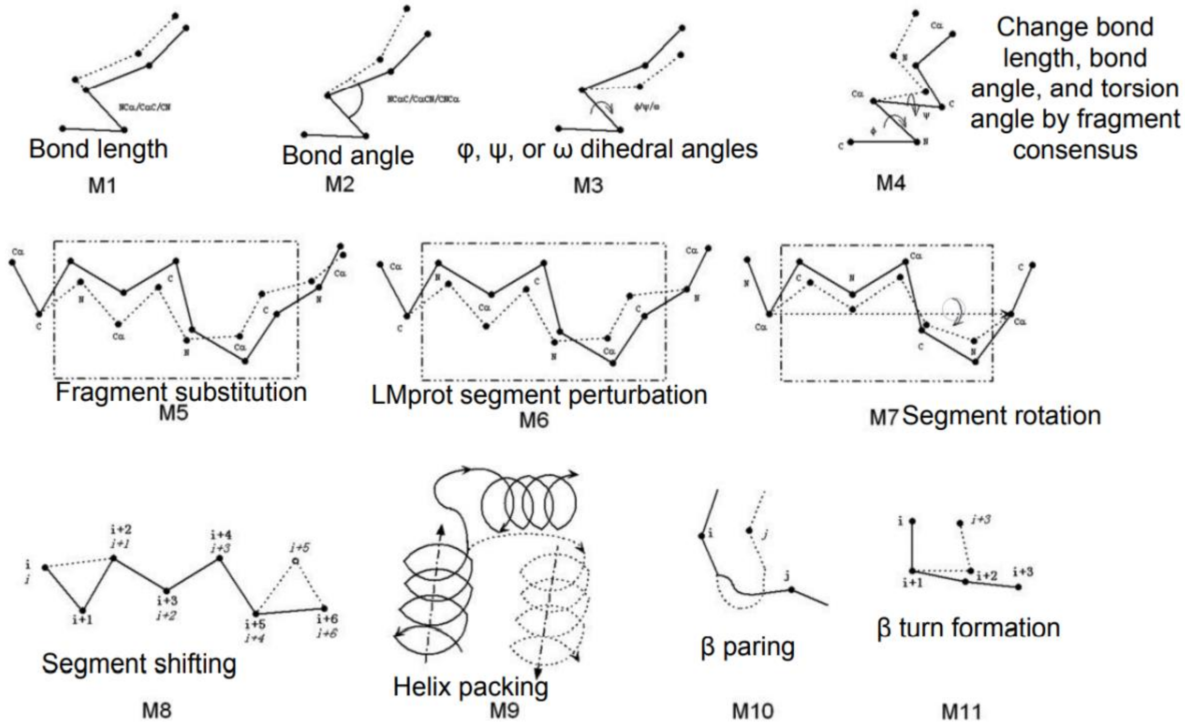

Figure S16: Eleven local movements in the C-QUARK REMC folding simulations. These movements can further be divided into three levels: residue level (M1–M4), segmental level (M5–M8), and topology level (M9–M11). Movements M1, M2, and M3 randomly change one bond length, bond angle, and torsion angle of a randomly selected residue. Movement M4 substitutes these three parameters in the selected residue by the clustered values for this residue which most frequently occur in the template fragments at the position. Movement M5 substitutes one fragment in the decoy by another one randomly selected from the position-specific fragment structures. Movement M6 first randomly changes the positions of the backbone atoms in a selected segment and then tries to restrict all the bond lengths and bond angles within the physically allowable region. Movement M7 rotates the backbone atoms of a randomly selected segment around the axis connecting the two ending Ca atoms. Movement M8 shifts the residue numbers in a segment forward or backward by one residue, which means the coordinates of each residue are copied from their preceding or following residue in the segment. In movement M9, one helix is moved closer to another one. In a similar way, one β-pair is formed in movement M10. Movement M11 tries to form a β-turn motif for every 4-mer segment along the query sequence.

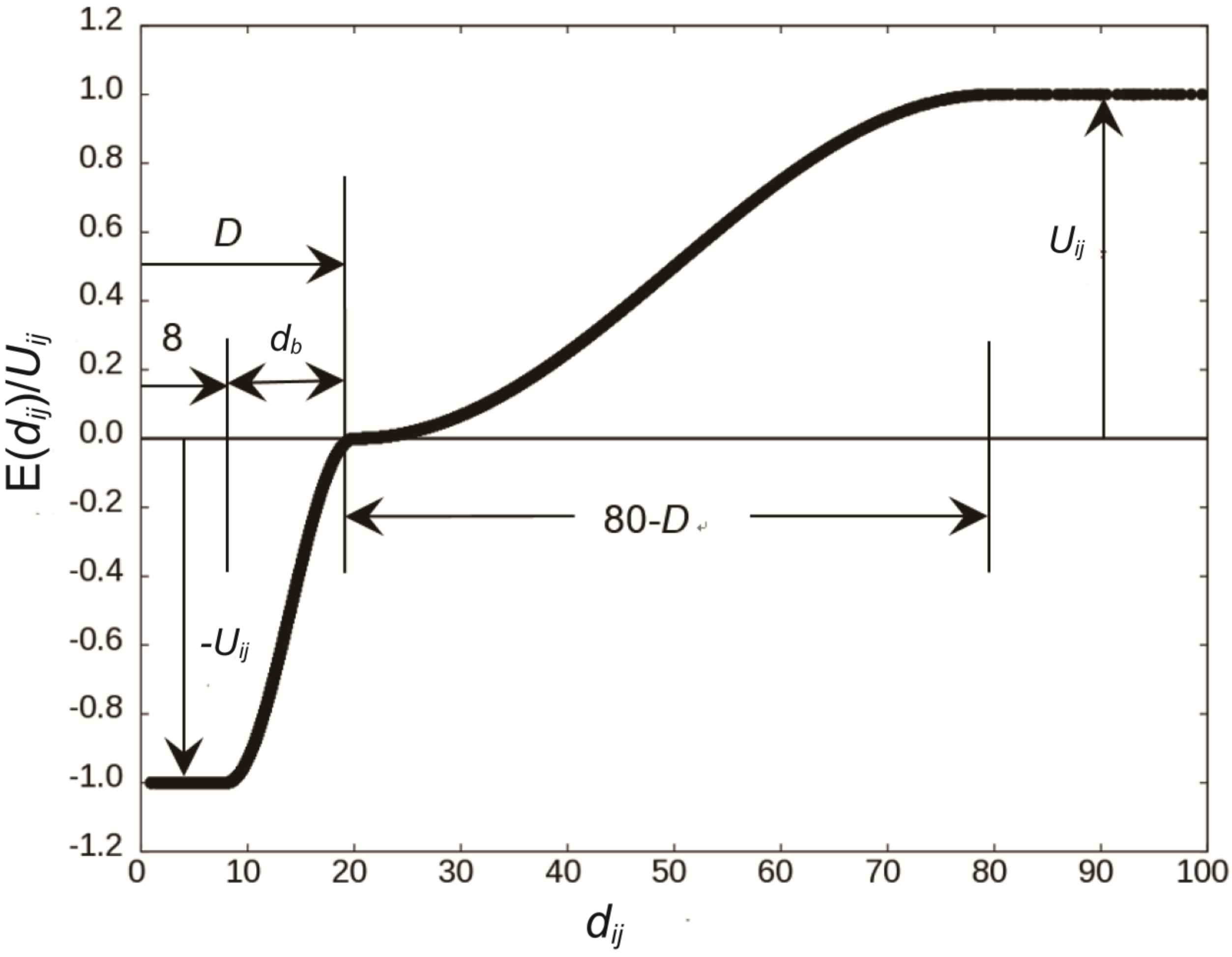

Figure S17: A schematic of the contact potential, $E_{con}(d_{ij})$, for a contacting residue pair i and j as defined in Eq. (1). Here, d_b is the width of the first well, which was tuned based on the training proteins (Dataset S1), and U_{ij} is the depth of the energy potential that is proportional to the confidence score of the predicted contact between the residue pair i and j (Eq. S4). d_{ij} is the C_β distance between the residue pair. The units for all the distances are in Å.

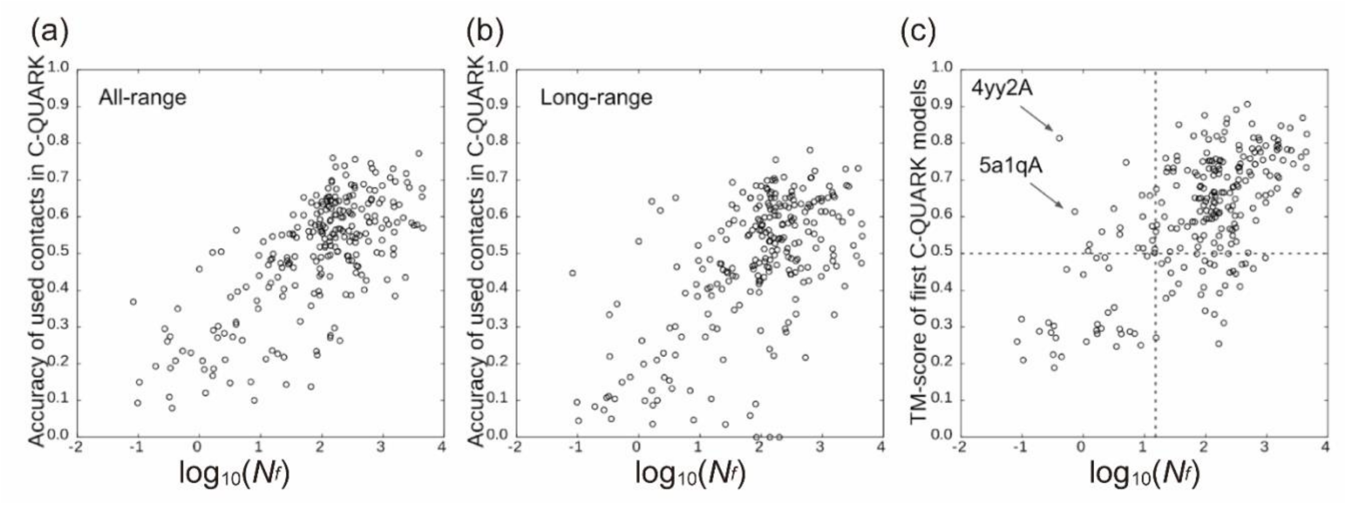

Figure S18: Effect of N_f on the accuracy of predicted contacts and C-QUARK model. Effect of N_f on the accuracy of predicted (A) all-range and (B) long-range contacts used in C-QUARK, where *i* and *j* refer to residue pairs in contact. (C) N_f versus TM-score. The horizontal dashed line indicates the TM-score cut-off of 0.5 beyond which models are considered to obtain similar folds as the corresponding native structures. The vertical dashed line indicates the N_f of 15, which is low. Out of 48 targets in the test set that have $N_f < 15$, C-QUARK can generate models with TM-score>=0.5 for 18 of them (i.e. 38% of the cases), including 4yy2A and 5a1qA, as highlighted with arrows, which have $N_f < 1$.

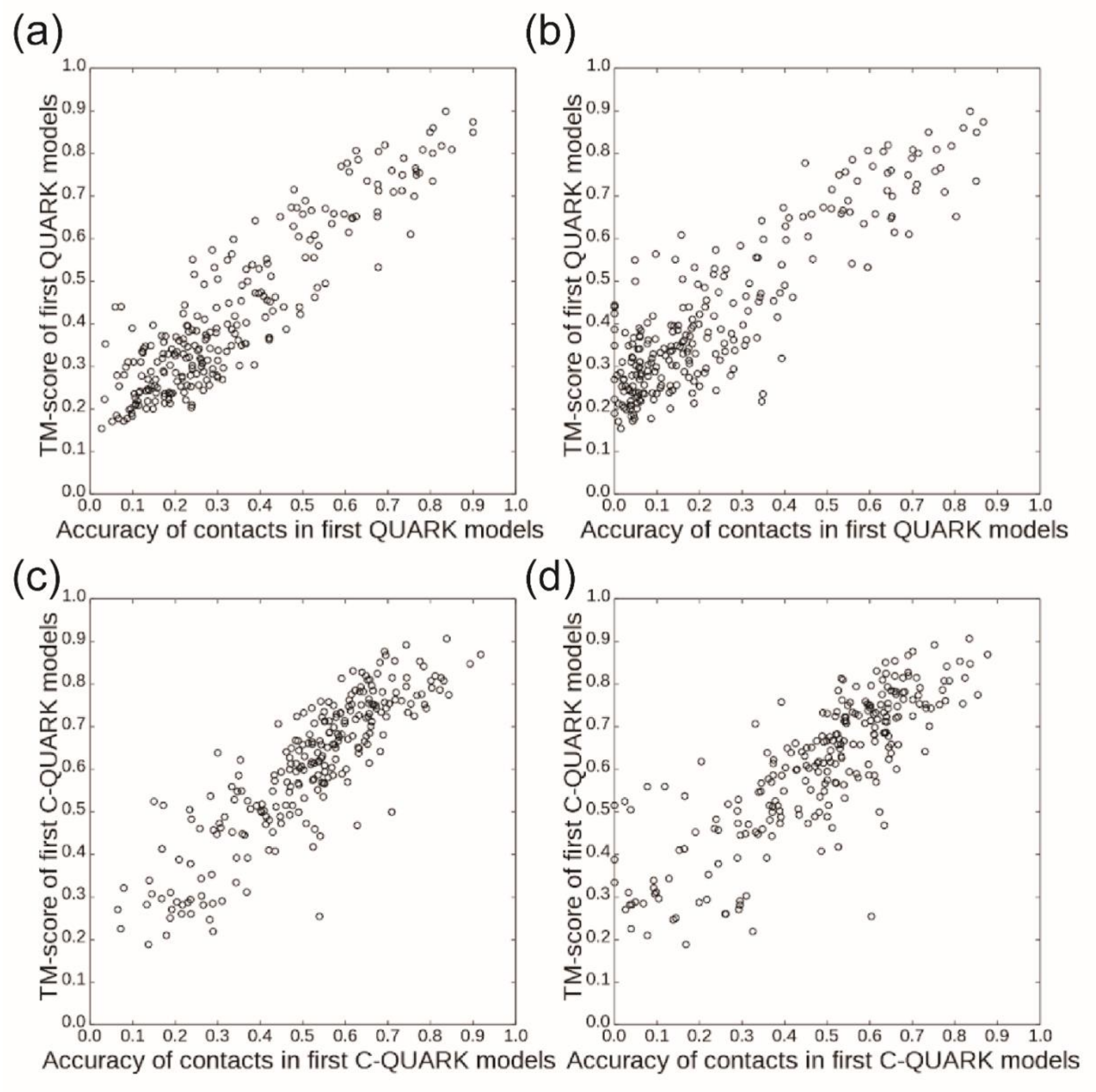

Figure S19: Relationship between TM-score and the accuracy of contacts for modeling. Relationship between TM-score and the accuracy of (A) all-range contacts and (B) long-range contacts for QUARK modeling. The PCCs are 0.904 and 0.877, respectively. Similar strong correlations are also shown for C-QUARK models in (C) and (D), respectively, where the corresponding PCCs are 0.868 and 0.824.

SUPPLEMENTARY REFERENCES

1. He BJ, Mortuza SM, Wang YT, Shen HB, Zhang Y. NeBcon: protein contact map prediction using neural network training coupled with naiive Bayes classifiers. *Bioinformatics* 33, 2296-2306 (2017).
2. Li Y, Yu D, Zhang Y. ResPRE: High-accuracy protein contact map prediction by integrating precision matrix with deep residual neural networks. (2018).
3. Jones DT, Kandathil SM. High precision in protein contact prediction using fully convolutional neural networks and minimal sequence features. *Bioinformatics*, bty341-bty341 (2018).
4. Liu Y, Palmedo P, Ye Q, Berger B, Peng J. Enhancing Evolutionary Couplings with Deep Convolutional Neural Networks. *Cell Syst* 6, 65-74 (2018).
5. Adhikari B, Hou J, Cheng J. DNCON2: improved protein contact prediction using two-level deep convolutional neural networks. *Bioinformatics*, (2017).
6. Buchan DWA, Jones DT. Improved protein contact predictions with the MetaPSICOV2 server in CASP12. *Proteins-Structure Function and Bioinformatics* 86, 78-83 (2018).
7. Kamisetty H, Ovchinnikov S, Baker D. Assessing the utility of coevolution-based residue-residue contact predictions in a sequence- and structure-rich era. *Proceedings of the National Academy of Sciences of the United States of America* 110, 15674-15679 (2013).
8. Seemayer S, Gruber M, Soding J. CCMpred-fast and precise prediction of protein residue-residue contacts from correlated mutations. *Bioinformatics* 30, 3128-3130 (2014).
9. Kajan L, Hopf TA, Kalas M, Marks DS, Rost B. FreeContact: fast and free software for protein contact prediction from residue co-evolution. *Bmc Bioinformatics* 15, (2014).
10. Zhang Y, Skolnick J. Scoring function for automated assessment of protein structure template quality. *Proteins-Structure Function and Bioinformatics* 57, 702-710 (2004).
11. Xu JR, Zhang Y. How significant is a protein structure similarity with TM-score=0.5? *Bioinformatics* 26, 889-895 (2010).
12. Xu D, Zhang Y. Ab initio protein structure assembly using continuous structure fragments and optimized knowledge-based force field. *Proteins-Structure Function and Bioinformatics* 80, 1715-1735 (2012).
