# Supplementary material for: Improving fragment-based ab initio protein structure assembly using low-accuracy contact-map predictions
Source: Nat Commun. 2021 Aug 18;12:5011. doi: 10.1038/s41467-021-25316-w (PMC8373938; doi:10.1038/s41467-021-25316-w)
Supplement: Supplementary file 3 — Description of Additional Supplementary Files [file 41467_2021_25316_MOESM3_ESM.pdf]

Description of Additional Supplementary Files

File Name: Supplementary Data 1
Description: PDB IDs of 243 Training Proteins

File Name: Supplementary Data 2
Description: PDB IDs of 247 Test Proteins

File Name: Supplementary Data 3
Description: CASP IDs of 64 Targets from CASP13
