# Supplementary material for: Improving fragment-based ab initio protein structure assembly using low-accuracy contact-map predictions
Source: Nat Commun. 2021 Aug 18;12:5011. doi: 10.1038/s41467-021-25316-w (PMC8373938; doi:10.1038/s41467-021-25316-w)
Supplement: Supplementary file 4 — Supplementary Data 1 [file 41467_2021_25316_MOESM4_ESM.pdf]

Supplementary Data S1. PDB IDs of 243 Training Proteins

1cf7B 1ci4A 1cqkA 1d2sA 1dqtA 1dyoA 1e85A 1eaqA 1em8A 1eyhA 1f32A 1f35A 1g652 1h9dB 1hcxA 1idpA 1ifrA 1jh6A 1jr8A 1kafA 1kmvA 1kptA 1l4dB 1lktA 1lwbA 1mg4A 1n08A 1nxhA 1nz0A 1od3A 1oh0A 1pinA 1pm4A 1qc7A 1qmyA 1r77A 1r7lA 1r9fA 1r9wA 1rljA 1rocA 1rreA 1s7kA 1seiA 1sluA 1szhA 1tc5A 1ty4A 1ufyA 1utyA 1uwfA 1v54E 1vjlA 1vmhA 1vqoN 1vziA 1w2wB 1w4sA 1wn2A 1wpuA 1x91A 1xfsA 1xkpC 1xphA 1xqsC 1y96B 1yd9A 1ygtA 1ykuA 1z9lA 1zhvA 2a2kA 2anxA 2avuE 2bl2A 2bukA 2bzvA 2c5kT 2ccmA 2cfxA 2ciuA 2co3A 2covD 2d0oB 2dkoA 2dokA 2erbA 2f01A 2f6mA 2f6mB 2f9zC 2fhzA 2fipA 2fjrA 2ftrA 2g2sA 2ga1A 2gagD 2gd9A 2gpiA 2i6gA 2i9wA 2ia7A 2ielA 2ijlA 2il5A 2iqyA 2j1vA 2j97A 2jcqA 2jekA 2o1qA 2o3bB 2o5aA 2o6fA 2od5A 2ofcA 2ogfA 2oixA 2opcA 2p9rA 2phpA 2pr7A 2pv2A 2pwwA 2q37A 2q3tA 2q7aA 2qdlA 2qeuA 2qfeA 2qklA 2qq4A 2qsdA 2qudA 2rjiA 2rk3A 2sicI 2uvpA 2v33A 2v3sA 2v75A 2vgxA 2vh3A 2vwaA 2vx8A 2yx8A 2yxyA 2z0bA 2z0rA 2zayA 2zbcA 3bb6A 3besL 3bpoA 3bpqB 3bwuF 3c1qA 3c5xC 3cgiA 3cm1A 3crjA 3db3A 3di2A 3dkaA 3dmbA 3doeB 3dxoA 3ebtA 3ef8A 3eipA 3ejkA 3eytA 3f6cA 3feaA 3fh2A 3fh3A 3pviA S0304 S0314 S0319 S0321_2 S0350 S0353 S0356_3 S0361 S0382 T0212 T0239 T0242 T0397_1 T0405_1 T0443_1 T0443_2 T0465_1 T0476_1 T0482_1 T0496_1 T0510_3 T0513_2 T0531 T0544_1 T0544_2 T0547_3 T0547_4 T0548 T0549 T0550_1 T0550_2 T0551 T0552 T0553 T0555 T0556 T0557 T0562 T0564 T0565_1 T0569 T0571_2 T0572 T0574 T0576 T0579_1 T0579_2 T0581 T0602 T0604_1 T0606 T0608_1 T0612 T0618 T0621 T0624 T0628_1 T0628_3 T0630 T0637 T0639 T0643 T0806 T0824 T0837
