# Supplementary material for: Improving fragment-based ab initio protein structure assembly using low-accuracy contact-map predictions
Source: Nat Commun. 2021 Aug 18;12:5011. doi: 10.1038/s41467-021-25316-w (PMC8373938; doi:10.1038/s41467-021-25316-w)
Supplement: Supplementary file 5 — Supplementary Data 2 [file 41467_2021_25316_MOESM5_ESM.pdf]

Supplementary Data S2. PDB IDs of 247 Test Proteins

Hard targets (109)
1a34A 1c8zA 1ckqA 1eaqB 1f47B 1fiuA 1jiwI 1ku1A 1kx9A 1p5uB 1pucA 1s0pA 1sdiA 1sr8A 1uujD 1y9iA 1y9lA 1zkeD 2b9dA 2bz1A 2d68B 2db7A 2fmmE 2huhA 2inwA 2o4tA 2oy9A 2pv4A 2pyqA 2qf4A 2vnvB 2xhhA 2xrhA 2xvsA 2yjlB 3a5pA 3bpjC 3eloA 3g20B 3gf6A 3ie4A 3ijwA 3jsrA 3kutA 3lgbB 3n5bB 3nikA 3nj2B 3njhD 3nymA 3p9aA 3sviA 3teqB 3u43A 3utkA 3v0rA 3v68A 3x0tA 3zfuA 3zr8X 3zzpA 4acjA 4aupB 4d5rA 4eg9A 4f98A 4g7xA 4gdzA 4h4nA 4hwxA 4iabA 4il7A 4l3uA 4lqzA 4mkoA 4ng0A 4npxA 4o7kA 4oe9A 4oelB 4peuA 4qpoA 4rvqA 4tnnA 4uonA 4wlrB 4wt3A 4wyhA 4x33A 4xb4B 4xo1A 4yy2A 4zuyA 5a1qA 5aizA 5aotA 5b5eZ 5c2uA 5cwpA 5ey0B 5ezuA 5i8jA 5jdkA 5jjeB 5kouB 5lusA 5ly8A 5sybA 5txuA

Easy targets (138)
1dhnA 1dugA 1fasA 1fk0A 1gv2A 1i8fF 1jztA 1kdkA 1kg5A 1l9lA 1mfwA 1n12A 1oh4A 1roaA 1tg0A 1vc4A 1vjkA 1wadA 1wwcA 1xtmB 1xu1A 1yocB 1zeqX 1zuuA 2b6eD 2bayE 2bswA 2cjsA 2cz4A 2d7jA 2f60K 2nn5A 2nsfA 2ofyA 2p39A 2pc1A 2ph0B 2plrA 2pttA 2qmlA 2qnlA 2rk5A 2rldC 2v4xA 2vvwA 2vywA 2w7qA 2wfoA 2wtgA 2x5pA 2y8pA 2zozB 2zyzB 3aj4A 3apaA 3dd7A 3e8tA 3eo6A 3eoiA 3exnA 3fjsC 3g5tA 3h8uA 3hftA 3iqtA 3kdfC 3ke7B 3kmjA 3kq0A 3linA 3m86A 3mpcA 3mqoB 3n9uC 3neuA 3no2A 3nojA 3oq2B 3p8bB 3qooA 3qzbA 3ro3A 3sxyA 3t6rA 3urrA 3v1oA 3vp5A 3waqA 3wwpA 4ac1X 4bdxA 4cg3A 4doiA 4e40A 4e5rA 4eweA 4fbjA 4gcoA 4hczA 4hu2A 4i5qA 4k12A 4kypC 4lafA 4lxqB 4mwzB 4nj8A 4o1rA 4o9gA 4oieA 4ovrA 4pp4A 4ps6A 4q7qA 4qbsA 4r78A 4s36A 4wvrB 4xunA 4ybnA 4yfbG 4yfvA 5a5yA 5c50B 5cj3A 5d7oA 5ebgA 5einC 5f67A 5fzsA 5ht7A 5k21B 5kehA 5lw6A 5t5iP 5tk6A 5tqjA 5u35A

Classification of topologies in the test proteins
i) *Alpha*-proteins: At least one *alpha*-helix is present, and no *beta*-strand is available.
ii) *Beta*-proteins: *Beta*-strands are available, and no *alpha*-helix is present except 3_{10}-*helix*.
iii) *Alpha-beta* proteins: Both *alpha*-helices and *beta*-strands are available.
