# Supplementary material for: Improving fragment-based ab initio protein structure assembly using low-accuracy contact-map predictions
Source: Nat Commun. 2021 Aug 18;12:5011. doi: 10.1038/s41467-021-25316-w (PMC8373938; doi:10.1038/s41467-021-25316-w)
Supplement: Supplementary file 6 — Supplementary Data 3 [file 41467_2021_25316_MOESM6_ESM.pdf]

Supplementary Data S3. CASP IDs of 64 Targets from CASP13

T0949-D1 T0953s1-D1 T0953s2-D1 T0953s2-D2 T0953s2-D3 T0954-D1 T0955-D1 T0957s1-D1 T0957s1-D2 T0957s2-D1 T0958-D1 T0959-D1 T0960-D2 T0960-D3 T0963-D2 T0963-D3 T0964-D1 T0965-D1 T0966-D1 T0968s1-D1 T0968s2-D1 T0969-D1 T0970-D1 T0975-D1 T0979-D1 T0980s1-D1 T0981-D1 T0981-D2 T0981-D3 T0981-D4 T0981-D5 T0985-D1 T0986s1-D1 T0986s2-D1 T0987-D1 T0987-D2 T0989-D1 T0989-D2 T0990-D1 T0990-D2 T0990-D3 T0991-D1 T0992-D1 T0997-D1 T0998-D1 T0999-D2 T1000-D2 T1001-D1 T1005-D1 T1008-D1 T1009-D1 T1010-D1 T1011-D1 T1015s1-D1 T1015s2-D1 T1017s2-D1 T1019s1-D1 T1021s1-D1 T1021s2-D1 T1021s3-D1 T1021s3-D2 T1022s1-D1 T1022s1-D2 T1022s2-D1
