# Supplementary material for: Improving fragment-based ab initio protein structure assembly using low-accuracy contact-map predictions
Source: Nat Commun. 2021 Aug 18;12:5011. doi: 10.1038/s41467-021-25316-w (PMC8373938; doi:10.1038/s41467-021-25316-w)
Supplement: Supplementary file 7 — Reporting Summary [file 41467_2021_25316_MOESM7_ESM.pdf]

nature portfolio

Corresponding author(s): Yang Zhang

Last updated by author(s): Jul 24, 2021

Reporting Summary

Nature Portfolio wishes to improve the reproducibility of the work that we publish. This form provides structure for consistency and transparency in reporting. For further information on Nature Portfolio policies, see our Editorial Policies and the Editorial Policy Checklist.

Statistics

For all statistical analyses, confirm that the following items are present in the figure legend, table legend, main text, or Methods section.

n/a	Confirmed	
☐	☒	The exact sample size (n) for each experimental group/condition, given as a discrete number and unit of measurement
☐	☒	A statement on whether measurements were taken from distinct samples or whether the same sample was measured repeatedly
☐	☒	The statistical test(s) used AND whether they are one- or two-sided<br>*Only common tests should be described solely by name; describe more complex techniques in the Methods section.*
☒	☐	A description of all covariates tested
☐	☒	A description of any assumptions or corrections, such as tests of normality and adjustment for multiple comparisons
☐	☒	A full description of the statistical parameters including central tendency (e.g. means) or other basic estimates (e.g. regression coefficient) AND variation (e.g. standard deviation) or associated estimates of uncertainty (e.g. confidence intervals)
☐	☒	For null hypothesis testing, the test statistic (e.g. F, t, r) with confidence intervals, effect sizes, degrees of freedom and P value noted<br>*Give P values as exact values whenever suitable.*
☒	☐	For Bayesian analysis, information on the choice of priors and Markov chain Monte Carlo settings
☒	☐	For hierarchical and complex designs, identification of the appropriate level for tests and full reporting of outcomes
☐	☒	Estimates of effect sizes (e.g. Cohen's d, Pearson's r), indicating how they were calculated

Our web collection on statistics for biologists contains articles on many of the points above.

Software and code

Policy information about availability of computer code

Data collection	The C-QUARK Online server and package is used for generating the models, where C-QUARK is available at https://zhanggroup.org/C-QUARK/ and https://github.com/jlspzw/C-QUARK.
Data analysis	R 4.0.3; CNS (CONFOLD) v1.0; DConStruct v1.0; trRosetta v1.0; MODELLER v9.21; NeBcon v1.0; ResPRE v1.0; DeepPLM v1.0; DeepCov v1.0; Deepcontact v1.0; DNCON2 v1.0; MetaPSICOV2 v1.0; GREMLIN v2.01; CCMpred v1.0 and FreeContact v1.0.21.

For manuscripts utilizing custom algorithms or software that are central to the research but not yet described in published literature, software must be made available to editors and reviewers. We strongly encourage code deposition in a community repository (e.g. GitHub). See the Nature Portfolio guidelines for submitting code & software for further information.

Data

Policy information about availability of data

All manuscripts must include a data availability statement. This statement should provide the following information, where applicable:

- Accession codes, unique identifiers, or web links for publicly available datasets
- A description of any restrictions on data availability
- For clinical datasets or third party data, please ensure that the statement adheres to our policy

We provide the accession codes list in the "Supporting Information" document, where all data can be downloaded from the PDB database using the provided accession codes. In addition, the entire dataset can be downloaded at https://zhanggroup.org/C-QUARK/ or https://github.com/jlspzw/C-QUARK.

Field-specific reporting

Please select the one below that is the best fit for your research. If you are not sure, read the appropriate sections before making your selection.

☒ Life sciences ☐ Behavioural & social sciences ☐ Ecological, evolutionary & environmental sciences

For a reference copy of the document with all sections, see nature.com/documents/nr-reporting-summary-flat.pdf

Life sciences study design

All studies must disclose on these points even when the disclosure is negative.

Sample size	The manuscript includes 243 targets in the training set, 247 targets in the testing set and 64 CASP targets. The CASP dataset is from the community-wide experiments and the other training and testing sets are collected from the PDB. We didn't use any particular statistical method to predetermine sample size, although for most of the statistical tests (i.e., t-test), n>=50 is enough to obtain a significant p-value. Thus, both our training and testing datasets are larger than 50. During the initial dataset construction, we selected around 5,000 single domain proteins from the PDB database, then ran LOMETS to decide the target type (i.e., Easy or Hard). Most of the proteins were Easy (template-based modeling) targets, leaving only around 240 Hard targets left. We randomly selected a comparable numbers of Easy targets (250) from the initial dataset of 5,000 proteins. This left us with 490 targets in total, which we randomly divided into two parts, the training dataset and the testing dataset. These datasets were used to benchmark the development of I-TASSER, QUARK, and C-QUARK.
Data exclusions	Proteins that were homologous to the test dataset were excluded from the template library to avoid homologous contamination. This criteria is widely used in protein folding studies. After data exclusion, all structures from the PDB library that shared over 30% sequence identity with the query protein were not selected as templates for modeling or used to define the target as a template-based modeling (TBM, Easy) target or free modeling (FM, Hard) target.
Replication	All results can be reproduced by our server and standalone package, or based on the information provided in SI.
Randomization	The training and testing proteins were selected randomly from the PDB, after excluding homologous proteins.
Blinding	The C-QUARK server was blind tested in CASP13 and the CASP13 results are reported in this article.

Reporting for specific materials, systems and methods

We require information from authors about some types of materials, experimental systems and methods used in many studies. Here, indicate whether each material, system or method listed is relevant to your study. If you are not sure if a list item applies to your research, read the appropriate section before selecting a response.

Materials & experimental systems

n/a	Involved in the study	
☒	☐	Antibodies
☒	☐	Eukaryotic cell lines
☒	☐	Palaeontology and archaeology
☒	☐	Animals and other organisms
☒	☐	Human research participants
☒	☐	Clinical data
☒	☐	Dual use research of concern

Methods

n/a	Involved in the study	
☒	☐	ChIP-seq
☒	☐	Flow cytometry
☒	☐	MRI-based neuroimaging
